# Advanced materials foresight: research and innovation indicators related to advanced and smart nanomaterials

**DOI:** 10.12688/f1000research.127810.1

**Published:** 2022-12-19

**Authors:** Lucian Farcal, Amalia Munoz Pineiro, Juan Riego Sintes, Hubert Rauscher, Kirsten Rasmussen

**Affiliations:** 1 European Commission, Joint Research Centre (JRC), Ispra, Italy; 2 European Commission, Joint Research Centre (JRC), Geel, Belgium

**Keywords:** Foresight, indicators, advanced materials, advanced nanomaterials, smart nanomaterials

## Abstract

**Background:** Advanced materials are most likely to bring future economic, environmental and social benefits. At the same time, they may pose challenges regarding their safety and sustainability along the entire lifecycle. This needs to be timely addressed by the stakeholders (industry, research, policy, funding and regulatory bodies). As part of a larger foresight project, this study aimed to identify areas of scientific research and technological development related to advanced materials, in particular advanced nanomaterials and the sub-group of smart nanomaterials. The study identified and collected data to build relevant research and innovation indicators and analyse trends, impact and other implications.

**Methods:** This study consisted of an iterative process including a documentation phase followed by the identification, description and development of a set of core research and innovation indicators regarding scientific publications, EU projects and patents. The data was extracted mainly from SCOPUS, CORDIS and PATSTAT databases using a predefined search string that included representative keywords. The trends, distributions and other aspects reflected in the final version of the indicators were analysed, e.g. the number of items in a period of time, geographical distribution, organisations involved, categories of journals, funding programmes, costs and technology areas.

**Results:** Generally, for smart nanomaterials the data used represent around 3.5% of the advanced nanomaterials data, while for each field analysed, they represent 4.4% for publications, 13% for projects and 1.1% for patents. The study shows current trends for advanced nanomaterials at a top-level information that can be further extended with sub-indicators. Generally, the results indicated a significant growth in research into advanced nanomaterials, including smart nanomaterials, in the last decade, leading to an increased availability of information.

**Conclusion:** These indicators identify trends regarding scientific and technological achievements and represent an important element when examining possible impacts on society and policy implications associated to these areas.

## Abbreviations

AdMa: Advanced materials

CLP: Classification, Labelling and Packaging of substances and mixtures (EU Regulation (EC) No 1272/2008)

CSS: Chemicals strategy for sustainability

CORDIS: Community Research and Development Information Service (by the European Commission)

EC: European Commission

EU: European Union

FAIR: Findable, accessible, interoperable and reusable

FP: Framework programme

IPC: International Patent Classification

KPI: Key Performance Indicator

NM – Nanomaterial

OECD: Organisation for Economic Co-operation and Development

OECD WPMN – OECD’s Working Party on Manufactured Nanomaterials

PATSTAT: The European Patents Office’s database on bibliographical and legal event patent data from leading industrialised and developing countries

REACH: Registration, Evaluation, Authorisation and Restriction of Chemicals (Regulation (EC) No 1907/2006)

REFIT: European Commission's regulatory fitness and performance programme

R&I: Research and innovation

SeTA: Semantic Text Analyser

SCOPUS: Elsevier's abstract and citation database

SSbD: Safe and sustainable by design

SSIA: Safer and Sustainable Innovation Approach

SNM: Smart nanomaterial

TIM: Tools for Innovation Monitoring

USPTO: United States Patent and Trademark Office

## Introduction

### Policy context

The European Union (EU) has adopted interconnected legislation to avoid trade barriers and ensure free movement of goods and people within the EU. The European Commission (EC) continuously evaluates whether EU legislation is meeting the needs of citizens and business through the European Commission's regulatory fitness and performance (REFIT) programme. ^1^ REFIT checks regulatory fitness and performance, aiming to ensure that EU legislation delivers results for citizens and businesses effectively, efficiently and at minimum cost, striving to make existing EU laws simpler and less burdensome to apply.

Among other things, EU legislation aims to ensure that chemicals placed on the market and the products in which they are incorporated, can be produced and used safely for humans and the environment. Safety of chemicals is addressed, among others, by two core pieces of legislation: the Regulations on Registration, Evaluation, Authorisation and Restriction of Chemicals (REACH) ^2^ and on Classification, Labelling and Packaging of substances and mixtures (CLP). ^3^ A vision for the chemicals of the future is to ensure that they are inherently safe and sustainable. ^4^ The latter requires, among others conditions, that they fit into a circular economy, ^5^ which is a concept aimed at minimising waste, reusing and recycling products, saving resources and preserving the environment. For the sake of regulatory preparedness, legislators desire to be able to predict the entry into the market of chemicals with tailor-made properties in order to understand if they would have any associated needs for updating the legislation and/or guidance related to safety and/or sustainability.

In order to achieve the goals set out in the Commission’s European Green Deal, ^6^ the Commission published a Chemicals Strategy for Sustainability (CSS) towards a toxic-free environment. ^4^ It is part of the EU’s zero pollution ambition, ^7^ which is a key commitment of the European Green Deal, which is also driving a New Industrial Strategy for Europe ^8^ that promotes responsible design and development of chemicals, materials and products. Safe and sustainable chemicals and materials ^9^^,^^10^ can help to reach these policy goals. ^11^ The Green Deal includes an action for boosting the investment and innovative capacity for production and use of chemicals that throughout their life cycle are safe and sustainable by design (SSbD).

One tool for predicting which chemicals enter the market is a foresight study. ^12^ Foresight studies explore the future of scientific and technological achievements and their potential impacts on society. They aim to identify the areas of scientific research and technological development that are most likely to bring about change and drive future economic, environmental and social benefits. Among others, foresight builds on indicators. An indicator is a quantitative or a qualitative measure derived from a series of observed facts that can reveal relative positions, e.g. at given regular intervals, and it can point to the direction of change across different units and through time. ^13^^,^^14^

According to the Commission’s 2021 Strategic Foresight Report, ^15^ the EU is a strong player in terms of knowledge and innovation, providing almost 20% of the world’s total research and development, publications and patenting activity. The EU has e.g. the largest share of worldwide patent applications in advanced manufacturing technologies and the Internet of Things for mobility. ^16^ The EU is a technological champion in advanced manufacturing and materials, with its industry delivering many critical enablers to global production lines, as well as a leader in future smart and sustainable mobility and low-carbon technologies. ^15^^,^^17^

**Figure 1.**
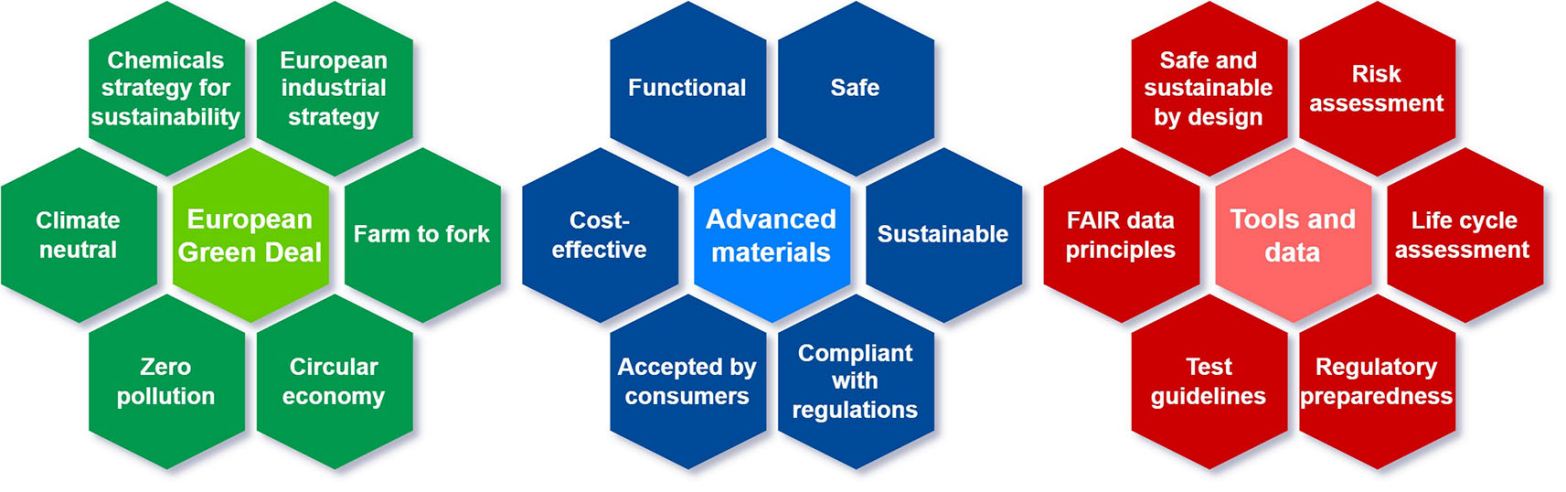
Illustration of the main policy areas (green) that steer the development of advanced materials (blue) and relevant tools (red).

Through its financial instruments and research and innovation programmes the EC supports research into and development of advanced materials for applications, for example in energy, construction, mobility, health, agriculture and the electronics sectors to deliver the green transition. ^4^^,^^8^ Regarding smart nanomaterials e.g. “ *It is expected that future research activities in the European Union will investigate whether the current approach to safe-by-design covers the dynamic features of smart nanomaterials too and, if not, how to adapt it and provide manufacturers and regulators with the appropriate tools for its implementation.*”. ^18^ Figure 1 illustrates how policy can steer the development of advanced materials towards safer alternatives and the associated tools required.

Thus, advanced materials are important drivers for the Green and Digital Transition, support the EU industrial recovery and can provide many economic, environmental and social benefits. However, as any other result of innovation, they may pose challenges (e.g. to ensure that they are safe and sustainable along their entire life cycle) that need to be timely addressed by regulators, in collaboration with other stakeholders. Some foresight regarding the kind of materials and areas of development of advanced materials seems appropriate as an initial step to anticipate those possible challenges. This should help both regulators and producers to be prepared to address them so that future advanced materials can deliver their maximum positive impacts to society.

In order to monitor progress, the EC intends to establish, in close cooperation with stakeholders, Key Performance Indicators (KPIs) to enable the measurement of the industrial transition towards the production of safe and sustainable chemicals as envisioned in the CSS. ^4^

### Advanced and smart (nano)materials

Advanced materials (AdMa) ^19^^–^^23^ generally mean materials that have novel or enhanced properties that improve performance in comparison to other materials already on the market (or the products and processes in which they are used for) and represent a broad class of materials that include semiconductors, biomaterials and nanomaterials. They can boost the transition to greener technologies, as they have improved characteristics and enhanced performance (which may include reduced environmental impacts), thus contributing to a more sustainable future. ^24^ The advanced materials “ *are associated with progressive technologies with the perspective to derive direct or indirect benefits in the form of highly specialised outcomes for multidisciplinary areas*”. ^25^ The new or enhanced properties (e.g. specific or improved performance) of AdMa are often determined by a combination of their chemical composition, physical properties, specific structures and higher complexity, often involving specific production processes. ^21^ Often today's advanced materials become tomorrow's standard materials, i.e. over time the currently new or improved properties and the resulting enhanced performance will become a common feature.

In the context of the OECD’s Working Party on Manufactured Nanomaterials (WPMN) ^26^ Steering Group on AdMa, they are understood as” *materials that are rationally designed to have new or enhanced properties, and/or have targeted or enhanced structural features with the objective to achieve specific or improved functional performance. This includes both new emerging manufactured materials, and materials that are manufactured from traditional materials. This also includes materials from innovative manufacturing processes, such as bottom-up approaches, that enable the creation of targeted structures from starting materials. It is acknowledged that what are considered to be AdMa will change with time*”. ^21^

Many sectors and applications rely on AdMa as the key to providing better solutions, including applications in safety and sustainability such as healthcare & medicine, construction, energy, transportation, home & personal care, packaging, agriculture, textiles, electronic appliances. ^27^^,^^28^ The “ *Materials 2030 Manifesto*” presents nine selected innovation markets and considers the European Green Deal and other policies that aim to create new value-chains.

It was proposed ^29^ that categorisation, or classification, of AdMa is possible taking into consideration:

• Functionality: active materials (smart, responsive, multifunctional, adaptive);
• Structure: structural materials (structured, multistructural, artificially structured);
• Manufacturing: advanced processes (controlled assembly of structures);
• Composition: composites, nanomaterials and bio-based materials.

Since the term advanced material is not univocally defined, the study mentioned above also aimed to characterise the use of the term 'advanced material' to obtain a reasonable distinction of advanced materials from other types of materials. Also, a set of criteria was described that could be applied to assess the ‘relevancy of advanced materials’. The proposed relevance assessment should allow the prioritisation of measures, e.g. with regards to the chemical safety. The four dimensions proposed for the relevance assessment includes: scientific (e.g. novelty of properties or the novelty of scale or combination of properties that advanced materials may have), economic and technical (e.g. their potential impact on technology development), hazard and risk (e.g. effect thresholds of different endpoints by exposure levels) and regulatory dimension (e.g. requirements for the generation and assessment of information on hazards and exposures as well as for the assessment of risks, coverage by the current legislation definitions and scope). In addition, a first description of identified advanced material clusters was performed. The outcomes of this analysis were presented in factsheets for each material, ^30^ which provide an overview of the characteristic properties of the identified advanced materials along with notes on the application range and potential risks and their regulatory status, as far as it can be anticipated.

Smart materials are materials that change their critical (functional) properties during use and activate specific functions upon exposure to external stimuli, which may come from their surroundings, for example a change in temperature, pH, light or contact with enzymes, to produce a dynamic and at times reversible change. ^31^ Examples include sensors and targeted delivery systems (or carriers), which are already used in medical products, cosmetics and electronics, and furthermore R&D (research and development) applications are under development for e.g. agriculture, food, and packaging. ^32^ An overview of the different types of smart materials available is provided in the ‘Smart Materials Books Series’ published by the Royal Society of Chemistry ^33^ since 2012. Currently it is a collection of forty-three (43) books, including ten book titles referring to nanomaterials.

### Study objectives

The aim of this project, as part of a larger foresight study, is to identify areas of scientific research and technological development related to advanced materials, in particular advanced nanomaterials and the sub-group smart nanomaterials that are most likely to bring changes and drive economic, environmental and social development and benefits for the future. It should lead to insights into trends of future applications and support the anticipation of possible regulatory challenges. It should be noted that several players (e.g. industry, policy makers, funding bodies, researchers) could equally benefit from these outcomes.

**Figure 2.**
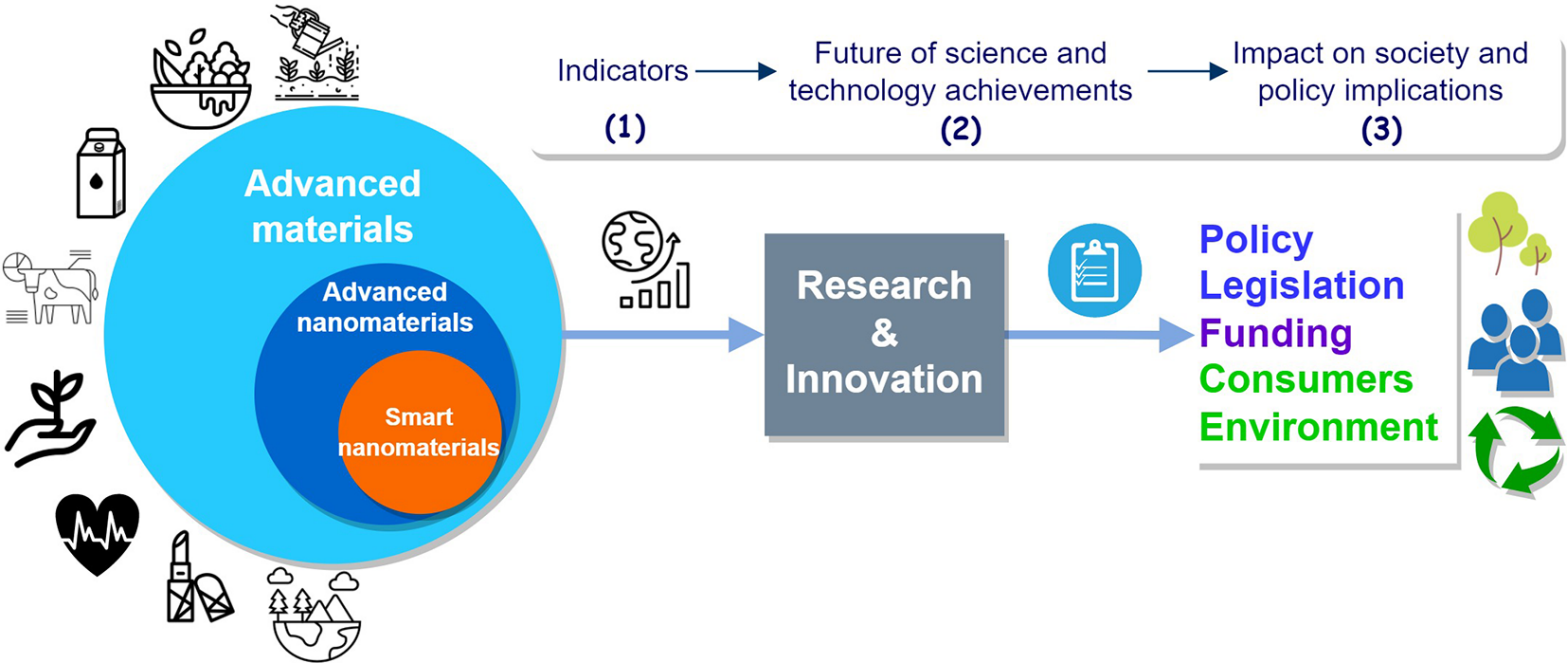
Schematic representation of the study objectives.

This study identifies and collects data in order to firstly build relevant indicators, and secondly to use the indicators to analyse the trends, impact and other implications, see Figure 2. This study addresses the objectives below sequentially:

(1) Identify and analyse key research and innovation indicators;
(2) Identifying trends and their drivers by analysing scientific publications, projects and patents on advanced nanomaterials, in an attempt to look into the future of scientific and technological achievements for advanced nanomaterials;
(3) Examine possible impacts on society and policy implications associated to advanced nanomaterials.

In order to achieve these objectives, several key questions need to be answered, e.g. what are the current trends related to the development of advanced nanomaterials, which are the dominant sectors, organisations and countries that develop advanced materials, or what are the (current or future) policy actions that aim to resolve the upcoming challenges related to the advanced nanomaterials?

## Methods

In order to achieve the goals of the study, a methodology for creating indicators was established. As a starting point, the study analysed relevant policy documents, especially those related to the European Green Deal ^6^ and its priorities, in order to identify some of the already defined directions towards safe and sustainable advanced materials. The study then examined and analysed research and innovation trends (e.g. publications, projects and patents) in these areas in relation to the European policies or legislation.

The methodology applied to establish research and innovation indicators related to advanced (nano) materials is described below. It consists of an iterative process that involves a background analysis of policy documents in the context of the topics covered by the study (e.g. advanced materials, advanced nanomaterials and smart nanomaterials) and a preliminary collection of information. Once the context is established, the methodology includes the identification and definition of indicators and further indicators development that involves data collection and analysis, by defining first the keywords for data search and the use of several tools for data extraction and analysis from databases. The methodology presented can be applied to any (sub) group of materials, for example smart nanomaterials, multicomponent nanomaterials, etc. by defining first an appropriate set of keywords to be included in the search strings.

Following the analysis of relevant policy and technical documents, a list of keywords and the desired indicators were created. This was an iterative process that aimed to identify, describe and collect data that is used to analyse trends and perspectives for advanced nanomaterials.

### Background analysis and preliminary information collection

Besides the analysis of specific technical reports on advanced materials (as shown in the introduction section), the methodology consisted also of identification and analysis of several policy documents, foremost the European Green Deal policy documents (e.g. EC Communications regarding the EU Green Deal, the new Industrial Strategy for Europe, the new Circular Economy Action Plan, the Farm to Fork Strategy and the Chemicals Strategy for Sustainability). This preparatory phase generated a preliminary list of keywords. Therefore, the set of European Green Deal policy documents, ^4^^–^^8^^,^^34^^,^^35^ described in the introduction, were consulted and analysed in order to refine the objectives of the study, as well as to support the keywords definition for developing indicators, as shown below. The analysis was performed, first by extraction of terms (words) and their frequency used in these policy documents, using KNIME, a free and open-source tool. ^36^ The *terms related to the materials, sectors, safety and sustainability* or *regulation and policy* (about 190 terms) were then selected and used to further refine and enrich the set of keywords for the R&I indicators data collection. The analysis included also the ranking of these terms based on their frequency in the policy documents ( *data not shown*).

**Figure 3.**
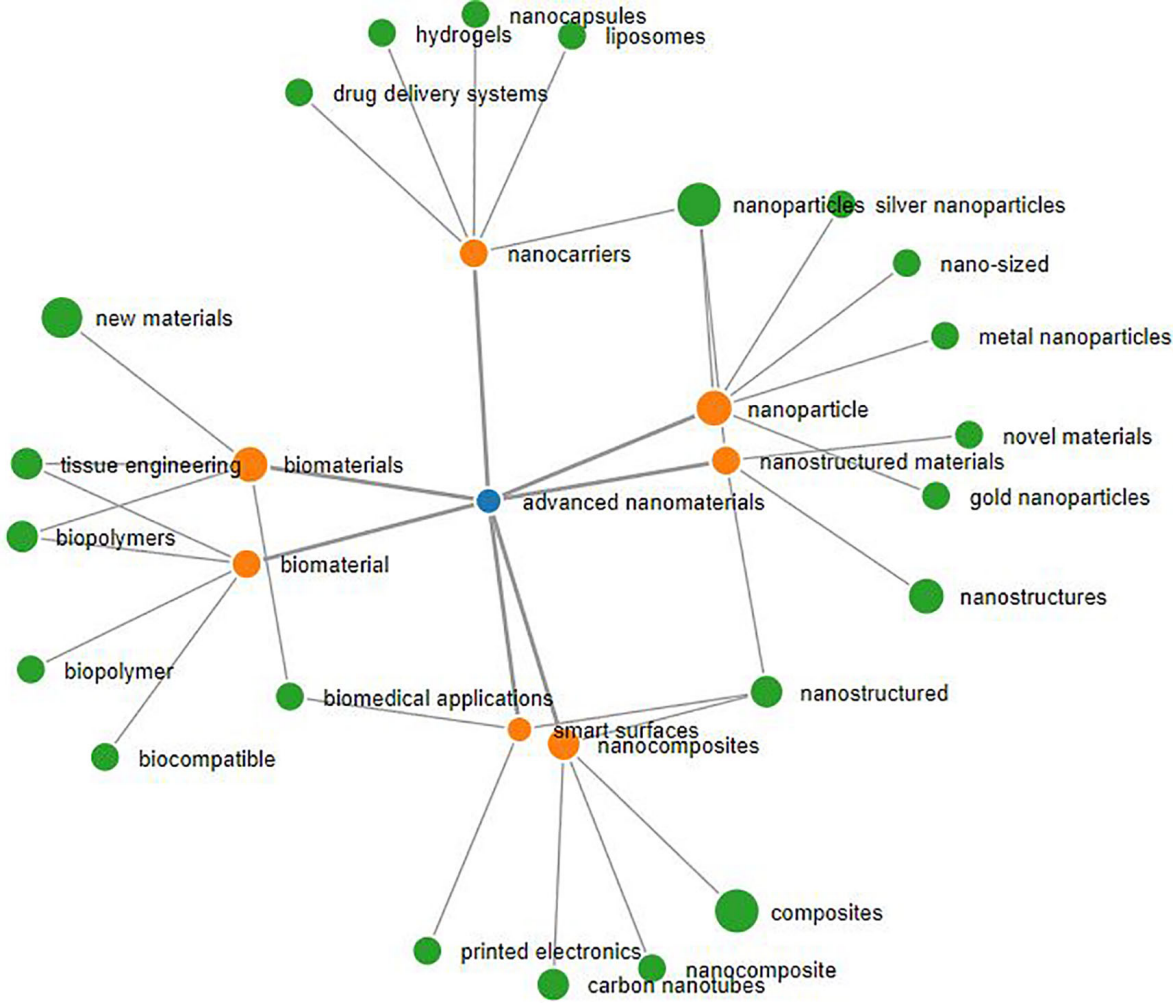
The network of most similar concepts for the term advanced nanomaterials (blue dot) extracted using SeTA tool (the concepts in orange are those closer to the term advanced nanomaterials, e.g. with a SeTA score equal or above 0.34, while in green are the concepts with a lower score). The size of the dots reflect the number of documents found by the tool e.g. advanced nanomaterials (blue dot) = 56 documents, biomaterials (orange dot) = 4761 documents, composites (green dot) = 17373 documents.

This background analysis was complemented with the application of open access tool Semantic Text Analyser (SeTA) “ *the information retrieval tool for policy makers*”. ^37^ SeTA applies advanced text analysis techniques to large document collections (e.g. from EUR-Lex, CORDIS, Data.europa.eu, Publications Office of the European Union), helping policy analysts to understand the concepts ( *defined by keywords, expressions and terms which are similar or related at the semantic level*) expressed in thousands of documents and to visualise the relationships between these concepts and their development over time. ^38^ For the current study, the tool was useful for automatically analysing the occurrence of several terms, such as *advanced nanomaterials* (see Figure 3 generated using SeTA tool), *advanced materials* or *smart materials*, which are already indexed in the SeTA database. Please note that some terms e.g. *new materials, biomaterials, functional materials, innovative materials, novel materials, nanostructured materials, composite materials, nanotechnology, nanostructured* and *composites*, were common to all three areas mentioned above. These results were used to further refine the list keywords to be used for the indicators.

### Identification and definition of indicators

In this step a preliminary list of indicators was prepared starting from terms identified by analysing the policy documents as described above. Subsequently, further analyses of their relevance and adequacy to the current study were carried out for the selection of provisional indicators that fit the purpose of the study. Their relevance to the advanced nanomaterials areas, as well as their ability to capture temporal, sectorial and/or geographical trends were considered, as briefly described in the following.

After the preliminary analysis, the fields (i.e. publications, projects, patents) to be searched in the study were defined and linked to advanced nanomaterials, including also a focus on smart nanomaterials.

**Figure 4.**
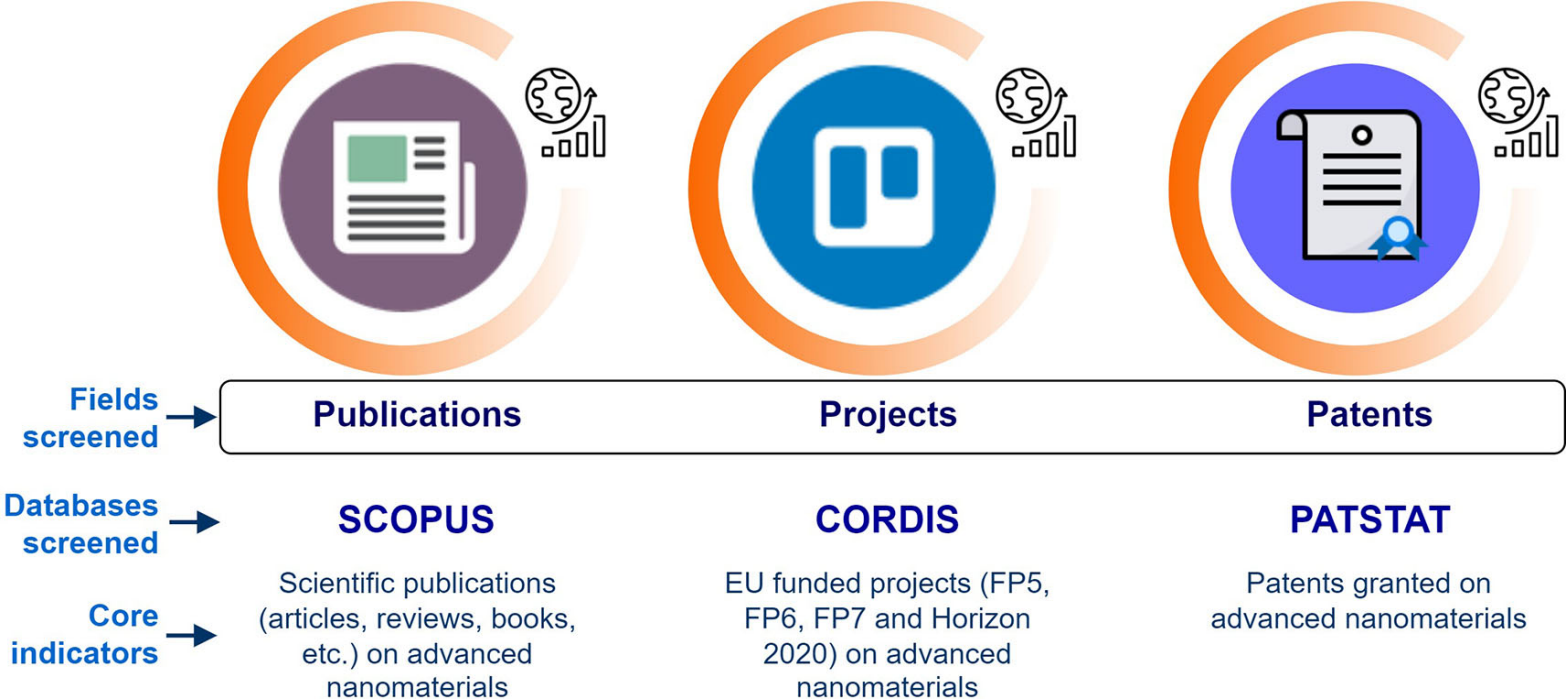
Fields and databases screened for creating the research and innovation indicators.

Then an initial list of core indicators relevant for the current study was defined which resulted from the investigation of research and innovation resources within the selected fields of scientific publications, EU projects and patents ( Figure 4). This was an iterative step, in which a brief description regarding the indicators’ title, relevance, data and possible sub-indicators was developed. The proposed sub-indicators focused on yearly trends, geographical distribution, organisations involved, journal categories for publications, costs of projects, keywords, etc.

### Data collection and analysis

In this step the extraction of data from public databases ( SCOPUS, CORDIS, PATSTAT and Data.europa.eu) using automatic data mining tools and analysis were performed using Tools for Innovation Monitoring (TIM). TIM is a tool developed by the EC’ Joint Research Centre (JRC) that regularly downloads the documents included in the above databases for further text mining. During the data mining process that extracts documents based on user-defined keywords combined in a search string, both the list of keywords and the search strings used for data collection were also refined. The collection of data, namely the number of publications, projects, etc. was done only by using the automatic tool TIM and based on the search strings mentioned later. No other manual (reviewers) screening was performed. For the indicators’ data, the screening did not include reports.

*Keywords definition*

The keywords definition was based on an *i*) analysis of scientific/technical reports on advanced and smart (nano) materials, *ii*) analysis of policy documents, as well as *iii*) the use of automatic tools for text analysis, as described above.

A set of keywords were defined in order to build comprehensive search strings and further use them to extract the data from the database. The sets of keywords cover the description of advanced (nano) materials ( *mainly functional and structural descriptors*), including few very specific descriptors for smart nanomaterials, see Table 1.

**Table 1.** List of final keywords describing the advanced materials including a sub-set of keywords specific to smart materials and nanomaterials.

Category	Keywords - functional, structural and other descriptors
Advanced materials	• active • adaptive • advanced • artificially structured • biobased • biomaterial • biomimetic • complex • composite • functional • hybrid • innovative • intelligent • multicomponent (multi-component) • multifunctional • multistructural • responsive • smart • stimuli responsive • structural • structured
Smart materials	• intelligent • smart • stimuli responsive ( *included also in the list above, as advanced materials descriptors*)
Nanomaterials	• nanoform • nanomaterial

The strings used for the searches were created in a way that the data extracted includes a combination between nanomaterials-related terms and the “ *advanced*” descriptors are shown in Table 1. Additional filters were applied, depending on the specificity of the indicator and data required (e.g. data only on publications, projects or patents), timelines (e.g. data only between 2011 and 2020 for publications) or type of applications. The Boolean operators AND, OR and NOT were used to combine them. The final strings applied for each case are shown in the results section, together with the analysis performed.

For nanomaterials-related terms, several options were tested during the study iterations. For example, the addition of other nano-related descriptors generated very large datasets (e.g. the addition of terms like “nano” or “nanoparticle” resulted in more than 400 000 results for publications, while the search using “nano*” generated more than 800 000 results because the use of wildcard ‘*’ generated data on all possible combinations of nano, and therefore gave less specific results. Most likely, the different options have an impact both on the amount and relevance of the data collected, and thus also on the specificity of the indicators. Finally, in order to generate a highly relevant set of results, which is representative for the topic covered by the study only the terms “nanoform” and “nanomaterial” were used in the final search string. In addition, by default TIM applies a stemming ^39^ process to the search terms entered by the user, in order to widen the set of matched documents (e.g. the term “technology” will match both “technology” and “technologies”).

Furthermore, a set of keywords to cover the uses of these types of advanced materials were defined. Without being complete, this list, which is based on the examples of applications described previously ^30^ with further additions, intends to allow the analysis and extraction of a representative set of data for the areas and sectors which use the advanced nanomaterials.

Finally, the selection of keywords followed an iterative approach in which a set of recent policy documents and scientific literature were consulted. ^18^^,^^29^^,^^30^^-^^35^

*Data extraction and analysis*

Based on the keywords, the search strings were defined for each individual indicator and used in the search engine for data extraction. The main tool used for data extraction was the TIM Technology Editor ^40^ that facilitates the access to data related to science and innovation and allows to create and visualise datasets about specific technological issues, that otherwise would have been carried out into the specific databases and manually combined. The search was performed in the fields title, abstract, author keywords and automatic keywords ^39^ of the documents, the automatic keywords were generated by TIM, which attributed a variable number of words (10-15) to each document. In our study TIM brought together datasets (exported as.xlsx files) from different sources regarding patents, scientific publications and EU grants: 1) peer-reviewed scientific publications from SCOPUS, 2) worldwide patent applications from PATSTAT and 3) projects funded by the EU's framework programmes for research and innovation (FP5 to Horizon 2020 [H2020]) from CORDIS, the EC’s Community Research and Development Information Service. For the latter, additional data files with detailed information on FP5, FP6, FP7 and H2020 projects were extracted directly from the European data portal and used for analysis, especially regarding the costs of the projects, and the data sources used were SCOPUS, CORDIS, PATSTAT and Data.europa.eu. The exact strings applied are listed in Table 2, therefore included a general search (in all 3 databases connected to TIM and also a separate search, publications, projects or patents. For the purpose of reproducibility, an alternative to the TIM Technology Editor used in this study users can access “ TIM Open Access”, a version of TIM without restrictions and that allows its users to perform searches and analysis on Open Access data. Therefore, it currently offers access to publications from Semantic Scholar, worldwide patent applications from PATSTAT, and projects EU funded projects from CORDIS. In addition, the same searches can be done in each of the databases separately, the results would then need to be combined manually. TIM facilitates the extraction of all together and the visualisation. Except Scopus, all are open access.

**Table 2.** Search strings used in the searches performed in TIM. The first string is the general final string, and the subsequent entries show the extension of each string for the three cases of publications, projects and patents.

Topic	Search string	Number of results	Databases searched by TIM
General search string *			
Advanced nanomaterials	topic:((nanoform OR nanomaterial) AND (active OR adaptive OR advanced OR "artificially structured" OR biobased OR biomaterial OR biomimetic OR complex OR composite OR functional OR hybrid OR innovative OR intelligent OR multicomponent OR multi-component OR multifunctional OR multistructural OR responsive OR smart OR "stimuli responsive" OR structural OR structured))	68,215	SCOPUS, CORDIS and PATSTAT
Smart nanomaterials ^‡^	topic:((nanoform OR nanomaterial) AND (intelligent OR smart OR "stimuli responsive"))	2,395 (3.5% of advanced nanomaterials)	
Additional queries applied for Publications ***			
Advanced nanomaterials	AND class: (conf OR boch OR review OR article) AND emm_year:[2012 TO 2021]	45,687	SCOPUS
Smart nanomaterials ^‡^	AND class: (conf OR boch OR review OR article) AND emm_year:[2012 TO 2021]	2,041 (4.4% of advanced nanomaterials)	
Additional queries applied for Projects *			
Advanced nanomaterials	AND class:euproject	563	CORDIS
Smart nanomaterials ^‡^	AND class:euproject	77 (13% of advanced nanomaterials)	
Additional queries applied for Patents **			
Advanced nanomaterials	AND class:patent	3700	PATSTAT
Smart nanomaterials ^‡^	AND class: patent	43 (1.1% advanced nanomaterials)	

Data retrieved:

* 2-Feb-2022;

** 21-Jun-2022;

*** 23-Jun-2022;

‡ subgroup of advanced nanomaterials.

**Figure 5.**
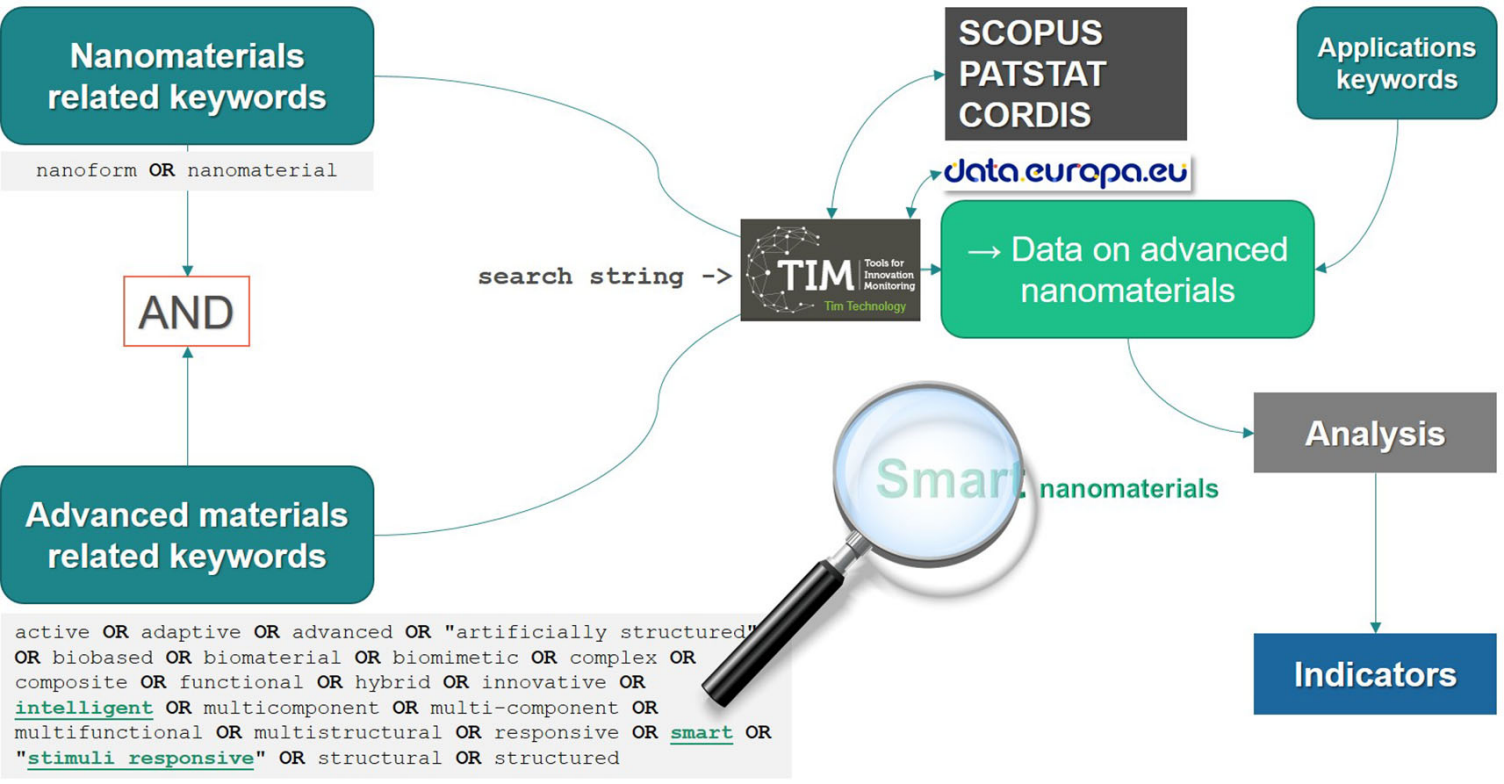
Data collection and analysis workflow.

The creation of the final version of the indicators followed the above process and workflow ( Figure 5) including some iterative refinement steps. First the collection of data using TIM was performed based on keywords related to advanced (including smart) nanomaterials description as well as keywords related to the application of these materials. The resulting data was analysed by looking at various aspects, like trends, distributions, and finally, the indicators were developed, including selecting how to visualise and report. The analysis included several aspects (e.g. for publications) and included indicators such as yearly distributions, categories of journals, author keywords or geographical distribution; for EU funded projects the analysis included aspects such as yearly distribution, value of projects, geographical distribution (e.g. countries, organisations); for patents the analysis looked into the yearly distribution, technology sub-areas or geographical distribution.

The figures included in the Results section were created with Microsoft Excel, while the maps were created with MapChart using the data extracted with the method presented above.

## Results

**Figure 6.**
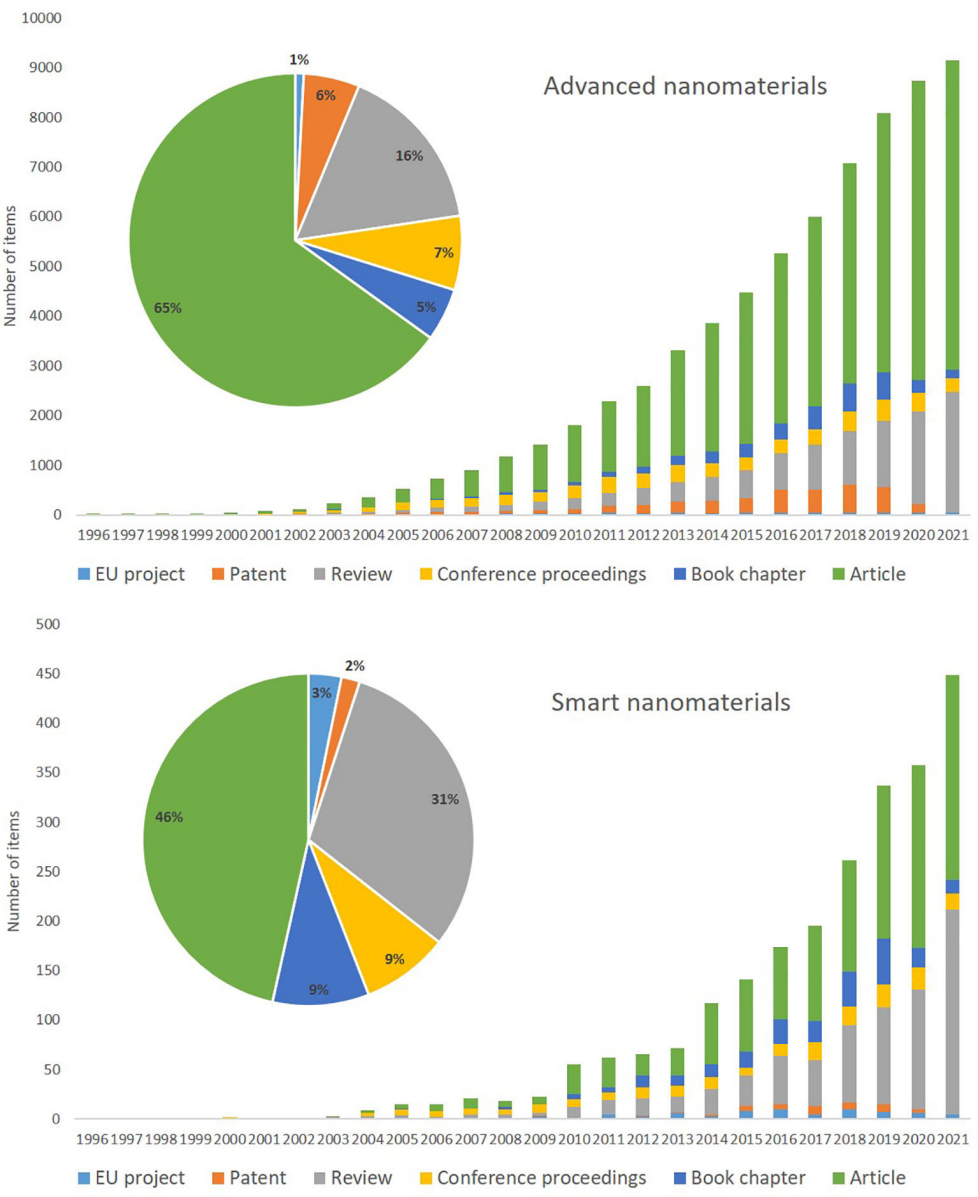
Amount and timeline for overall data for EU projects (number of projects and the year that the project started), patents and publications (articles, reviews, book chapters and conference proceedings) extracted using TIM on advanced nanomaterials (top) and smart nanomaterials (bottom). Please note that the scale of the y-axis, number of items, is different for advanced nanomaterials and smart nanomaterials.

The final search strings in TIM for “advanced nanomaterials” and for “smart nanomaterials” (see Table 2) and the data generated ( *from 1996 until the end of 2021*) ( Figure 6) were used as a starting point for the detailed analyses and to create the indicators for the three major areas screened in this study, i.e. scientific publications, EU funded projects and patents ( Table 3). Additional fields (e.g. timeframes, classes or topics) were added to these basic search strings, depending on the specificity of the analysis and the desired indicator. Generally, ‘node size’ data from TIM datasets was used for the analysis that represent the number of results (e.g. number of documents) for that specific indicator.

**Table 3.** List and description of indicators.

Title of core indicator	Short description	Data	Sub-indicators
Scientific publications on advanced nanomaterials	The indicator monitors the scientific publications, including articles, reviews, book chapters and conference proceedings on advanced nanomaterials, published yearly in the last decade (see Figure 7). This represents also a starting point for additional and more targeted sub-indicators and further detailed analyses performed on the data collected.	The number of publications per year, in the period 2012 to 2021, is extracted from the SCOPUS database using TIM. Additional datasets are represented by the keywords related to the publications, the location of the authors, the affiliation (organisations) of the authors and journal categories. A separate list of keywords is used to extract the types of applications and use of advanced nanomaterials.	• Distribution of publications to different categories of journals • Organisations involved in publications • Geographical distribution of publications • Distribution of publications per sector and type of applications • Trends in the keywords used in publications
EU funded projects on advanced nanomaterials	The indicator monitors the projects funded under the EU framework programmes FP5, FP6, FP7 and Horizon 2020 (see Figure 11). It looks into the yearly number and distribution of projects with more details on aspects such as cost, participating countries, etc.	The number of projects and the additional data (organisations, countries, keywords) are extracted from the CORDIS database using TIM. The files containing the total cost of projects are obtained from the European Data Portal. The data includes information on FP5, FP6, FP7 and Horizon 2020.	• Distribution of projects between the framework programmes and funding schemes • Costs of EU projects • Distribution of projects among EU member states • Organisations involved in EU projects
Patents granted on advanced nanomaterials	The indicator monitors the patents (the granting of a property right by an authority to an inventor) on advanced nanomaterials. The patent data are extracted from the database PATSTAT of the European Patent Office that contains patents from more than 90 patent authorities including all the major countries, therefore represents global data ( Figure 15).	The number of patents and additional data (organisations, countries, keywords, patent classification) are extracted from PATSTAT database using TIM.	• Distribution of patents to categories and technology areas • Geographical distribution of patents • Technology areas for the patents

In this section, the results for advanced nanomaterials, including smart nanomaterials (ca. 3.5% of the identified items for advanced nanomaterials), are analysed. In some cases, a specific evaluation of smart nanomaterials data was also included.

Figure 6 depicts the total number of documents per year for EU projects, patents and publications (articles, reviews, book chapters and conference proceedings) for advanced nanomaterials and smart nanomaterials (bottom); the extraction was performed with TIM and the search strings are indicated in Table 2.

As seen from Figure 6 (top) for advanced materials there is a steady increase in publications from 2001 (63 articles) to 2021 (more than 6200 articles), and with the rise in shared knowledge also the number of reviews increases. The book chapters appear from 2008 onwards and fluctuate a bit; they represent 5% of the total publications. The annual number of conference documents have remained at a fairly stable level; relatively they represent 7% of the total number of documents. The patents steadily increase from 6 in 2001 to around 500 in 2019 and then seem to fall again; this decrease may reflect the time needed to grant patents. The EU projects represent the number of projects that the EU has (co) funded via its research programmes and the year that the project started; the number of projects increases constantly until 2011 followed by a stable number of projects per year with an average of ~40 projects/year. A detailed analysis of the data collected for the three areas is presented in the sections below.

### Publications

The information extracted from SCOPUS using TIM covers data for the period 2012 until 2021 and includes peer-reviewed articles (70 %), reviews (18%), book chapters (6%) and conference proceedings (6%) addressing advanced nanomaterials; the percentages is the relative distribution of the publication categories. However, for simplicity, in most of the indicators below, we have merged this data in one item called ‘Publications’, unless otherwise stated. Overall, in this period there are 45,687 publications on advanced nanomaterials, of which 2,041 (4.4%) refer to smart nanomaterials. The final search string used for this search is shown in Table 2.

**Figure 7.**
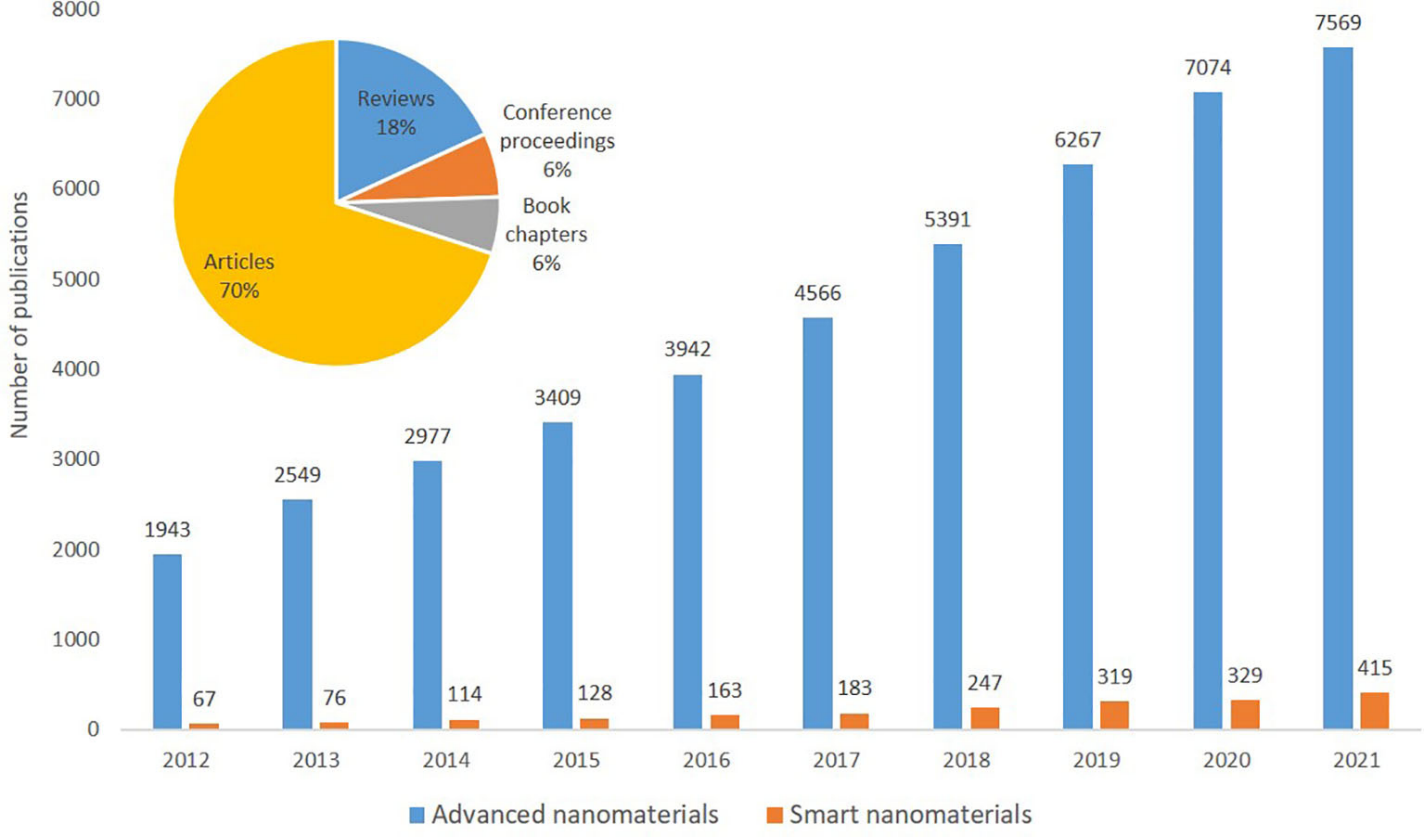
Yearly distribution of publications on advanced nanomaterials and smart nanomaterials in the period 2012-2021. The pie chart shows the percentage of different types of publications on advanced nanomaterials.

The extracted publications were analysed with regards to annual number of publications, see Figure 7. As expected, a significant increase in number of yearly publications was seen both for advanced materials (2012 = 1943 publications to 2021 = 7569 publications, i.e. an increase of 390 %) as well as for the sub-field smart nanomaterials (2012 = 67 publications to 2021 = 415 publications i.e. an increase of >600%).

When comparing with the publications on nanomaterials (data not shown), the trend is similar for the period analysed. The total number of publications on advanced nanomaterials (45,687) represent 56% of the total number of publications on nanomaterials (81,653) ^1^. Interestingly, this percentage increased constantly from 47% in 2012 to 58-59% after 2018. For the relative occurrence of the types of publications (articles, books, etc.), there are no significant differences between nanomaterials and advanced nanomaterials.

**Figure 8.**
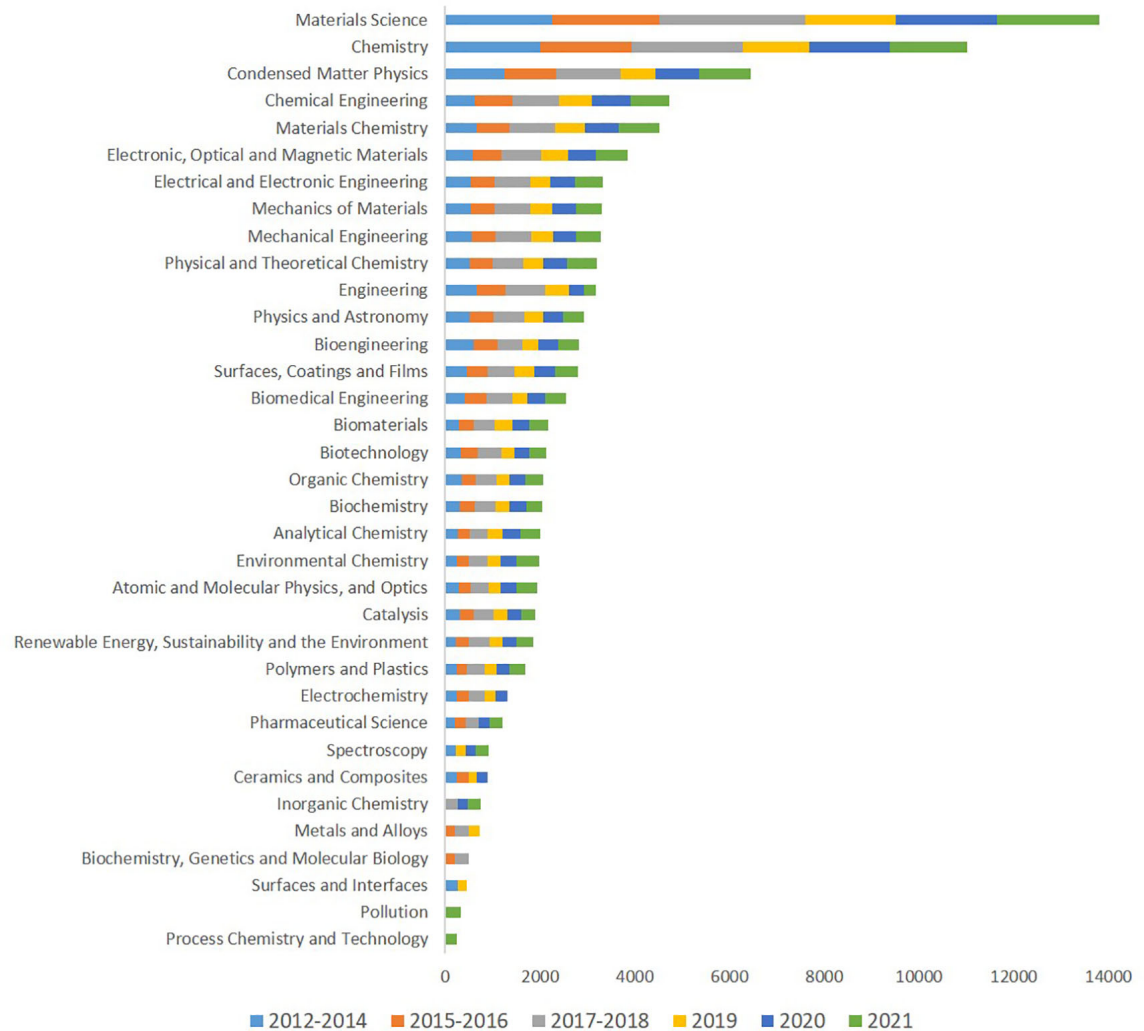
Distribution of publications on advanced nanomaterials to different categories of journals in the period 2012 to 2021.

Analysing how the publications on advanced nanomaterials are distributed over different categories of journals reveals the following pattern, see Figure 8., which shows the top thirty categories of journals ranked according to the number of publications and including the time distribution from 2012 to 2021. Material science and engineering are very well represented over the whole period, reflecting research into developing advanced nanomaterials and into understanding the properties and possible uses of them. Also, chemistry and surface chemistry are well represented, indicating that possibly the greater reactivity of nanosized materials is a sought-after property, this is supported by the appearance of Catalysis among these journal categories. These first categories are followed by journals from the biotechnology and bioengineering areas. Interestingly there are categories of journals (e.g. Pollution, Process Chemistry and Technology) in which the publications were listed more recently only (2021), while for other categories (e.g. Metals and Alloys, Biochemistry, Genetics and Molecular Biology, or Surfaces and Interfaces) there are no publications listed in the last years two years analysed (2020-2021).

**Figure 9.**
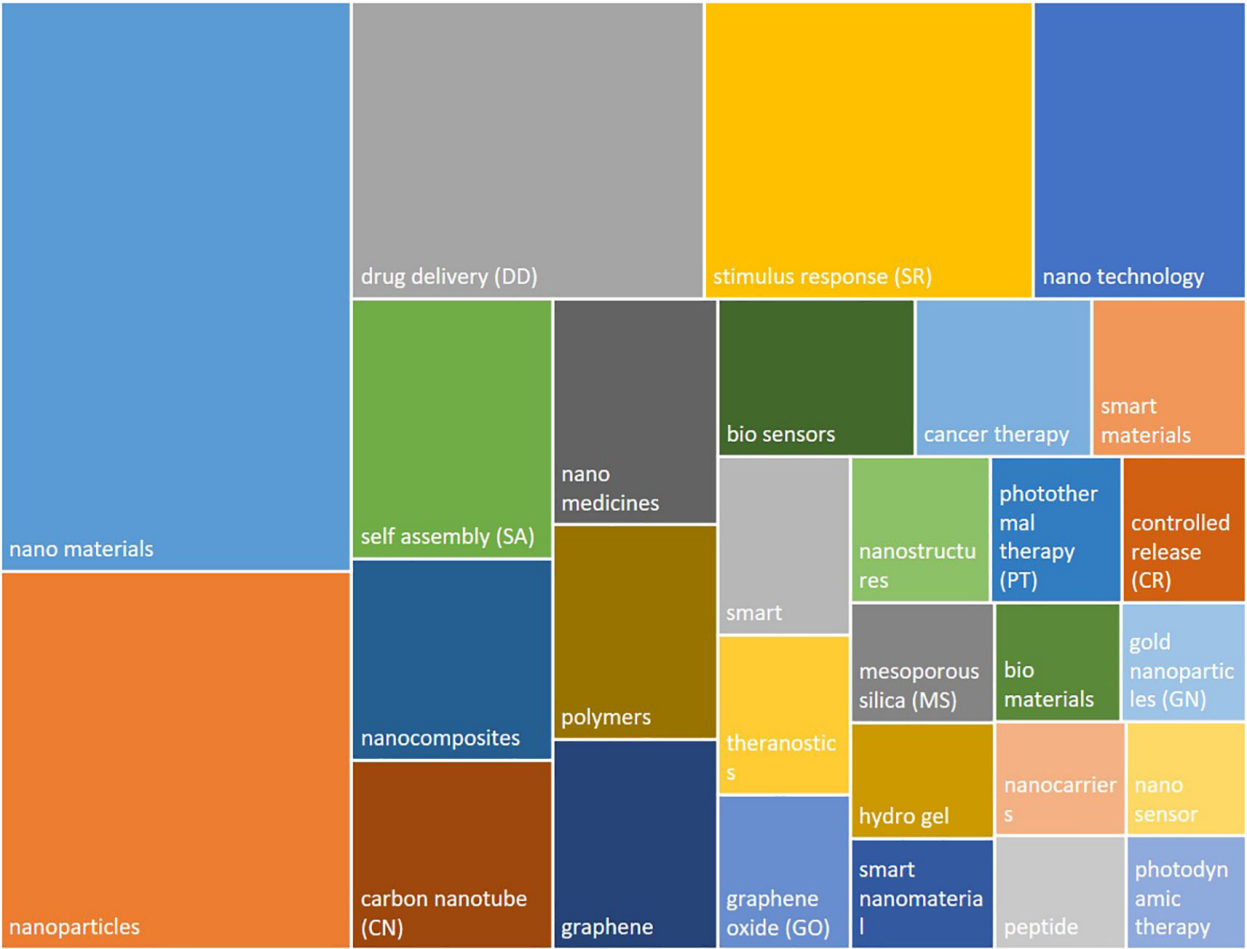
Most frequent author keywords used in the’smart nanomaterials’ publications. The box size reflects the frequency of use of the keywords.

An analysis of the most frequently used keywords is presented in Figure 9. It can be observed that most of the frequent terms refer to the material type (e.g. nanomaterials, nanoparticles, nanocomposites, carbon nanotube, graphene, polymer), to the functionality or specific property (e.g. drug delivery, stimulus response, self assembly, bio sensor, controlled release, mesoporous silica) or more general terms such as nanomedicine, nano technology, smart/smart material.

**Figure 10.**
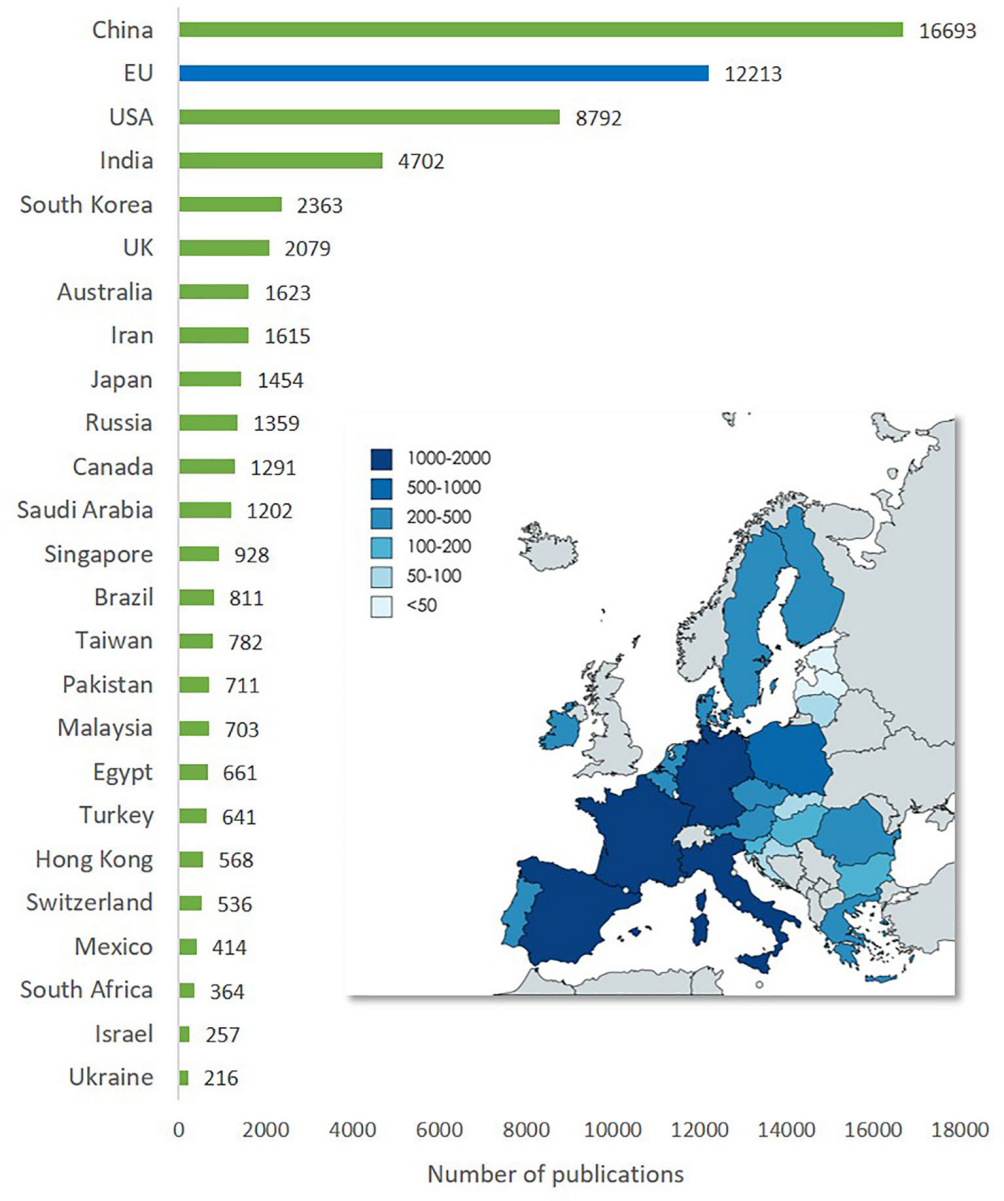
Geographical distribution of publications on advanced nanomaterials (top 24 countries and the EU). The map shows the distribution of publications on advanced nanomaterials in EU countries in the period 2012-2021.

The worldwide geographical distribution of publications on advanced nanomaterials for the top 24 countries and the EU ( Figure 10) shows that China is the country that has the highest number of publications (16,693), followed by the USA (8,793). The region of the EU is in between with 12,213 publications. In addition to the bar chart, the map inset of the EU illustrates the level of publications on advanced nanomaterials by the EU member states.

### Projects

**Figure 11.**
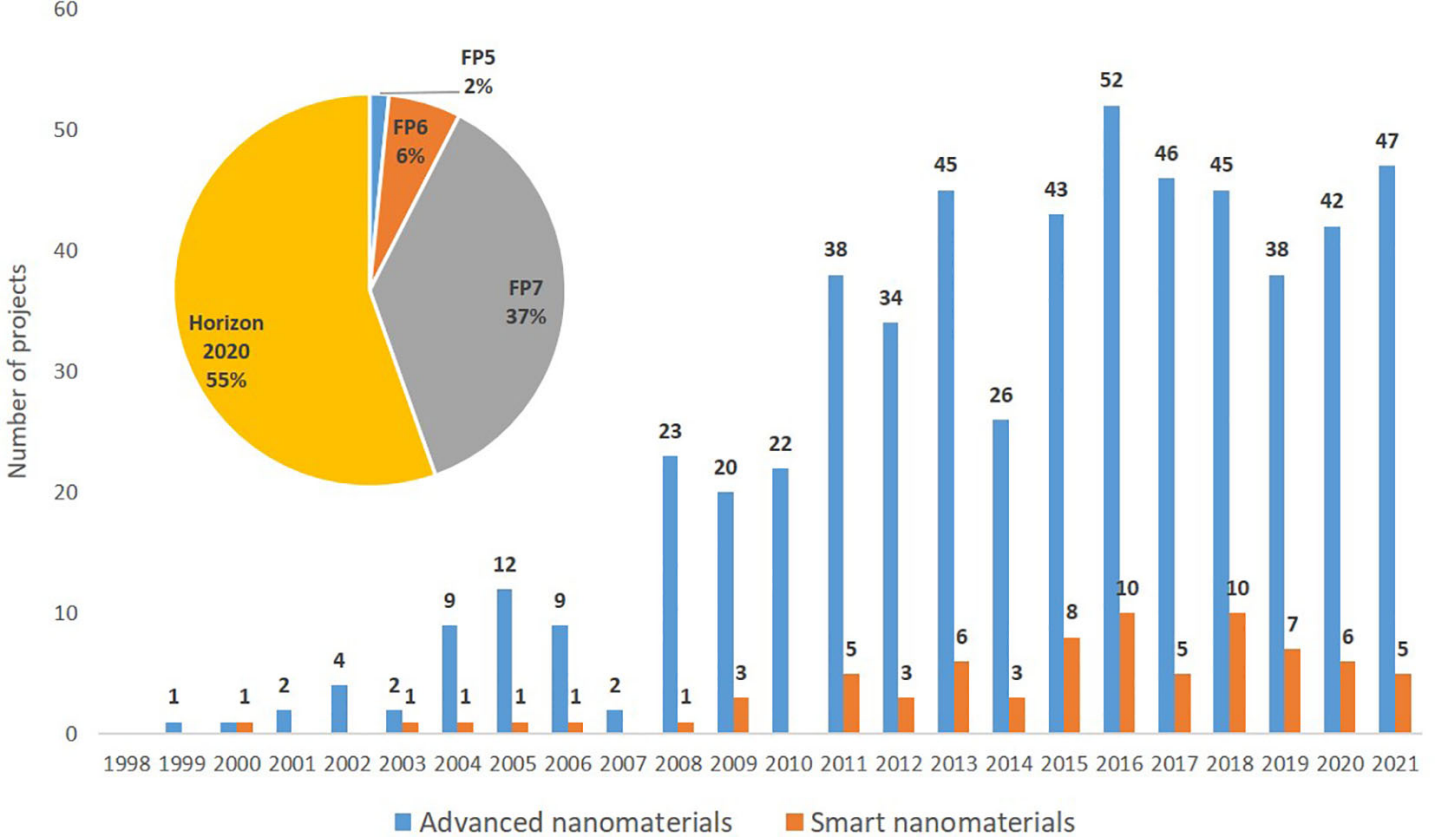
Yearly distribution (the bars) and percentage (the pie chart) of EU projects funded under FP5, FP6, FP7 and Horizon 2020 on advanced nanomaterials and smart nanomaterials.

**Figure 12.**
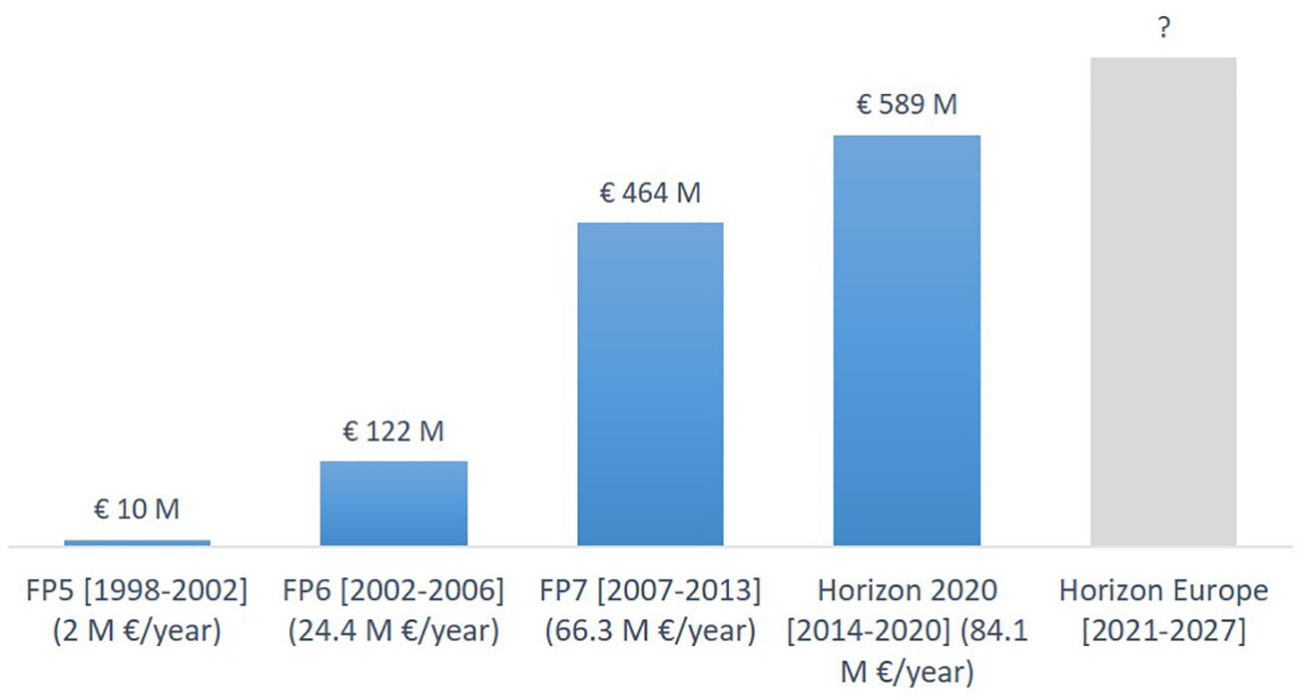
Value of EU projects (total value incl. partner contributions = € 1.18 billion) on advanced nanomaterials. To be noted that the duration of the framework programmes go from five years to seven years between FP6 and FP7.

The data on projects funded under the European framework programmes (FP5, FP6, FP7 and Horizon 2020) covering the period 1998 to 2021 was extracted from the CORDIS database using TIM, and 563 projects on advanced nanomaterials were identified, of which 77 (13.6%) are related to smart nanomaterials. The search string used is shown in Table 2, while the distribution of projects per year and framework programmes are illustrated in Figure 11 and Figure 12.

A significant increase in both number of projects and available funding is observed in the period 1999 (FP5) to 2020 (Horizon 2020). Furthermore, also the number of funded projects studying smart nanomaterials has increased from an average of 1 project per year in FP5 to 7 projects per year in Horizon 2020. As seen from Figure 11 the number of EU-funded projects has remained rather constant in the period 2011 to 2021. However, the project funding increased from €464 million under FP7 (2007 to 2013) to €589 million under H2020 (2014 to 2020), or in other words from €66.3 million/year to €84.1 million/year; see Figure 12.

**Figure 13.**
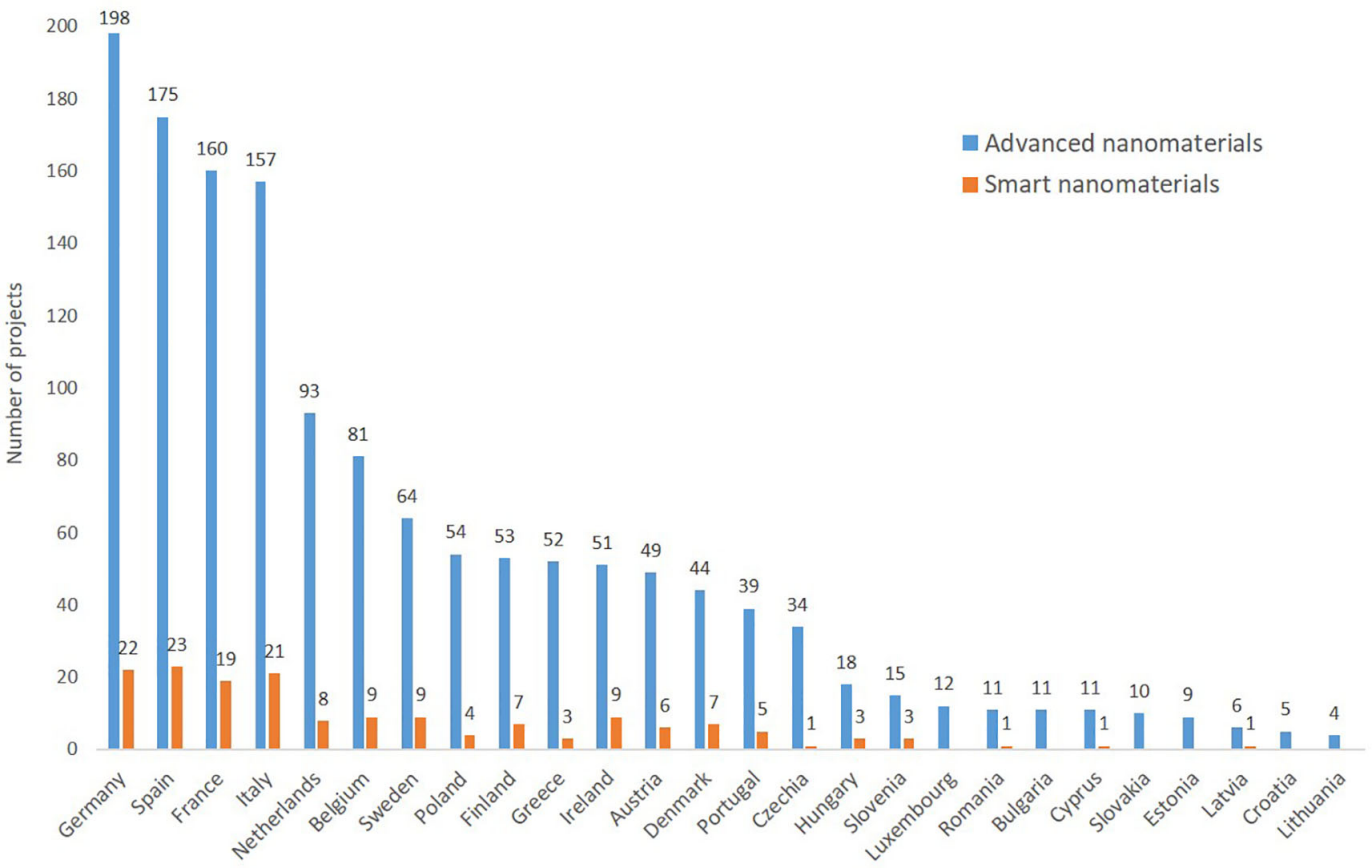
Participation by EU member states in EU funded projects (FP5 to Horizon 2020) on advanced nanomaterials and smart nanomaterials; Malta does not appear as they do not participate to any project in the areas analysed; the UK left the EU on 31 January 2020 and is not included here.

**Figure 14.**
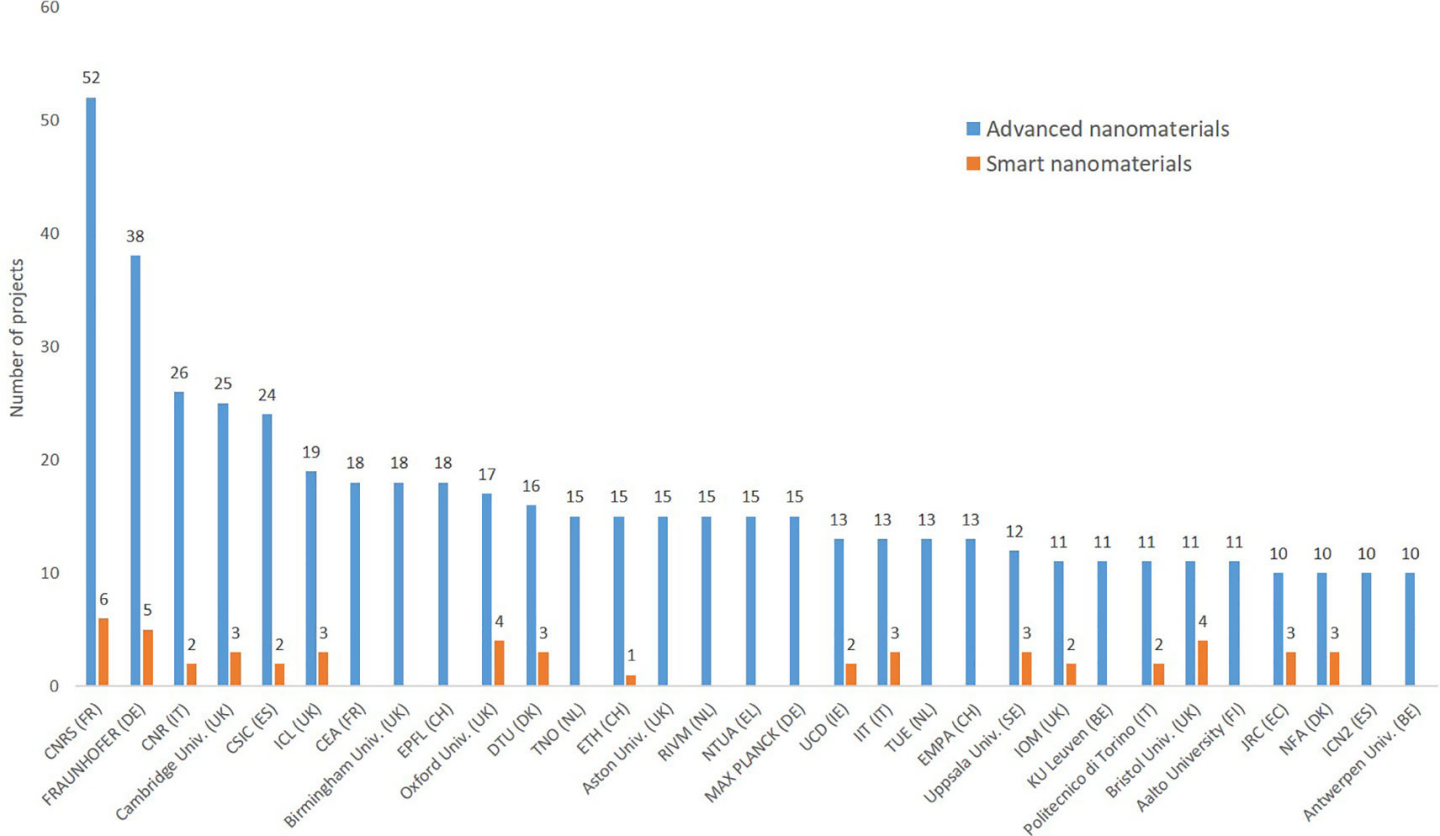
Top 25 organisations participating in EU projects related to advanced nanomaterials; as seen Switzerland is associated to the EU research programme, and the UK was deeply involved before Brexit, with 6 institutions among the top-25 institutions at EU level.

Regarding the geographical distribution among EU member states ( Figure 13) it can be seen that 26 of the 27 EU member states participate to the projects, and that the large member states participate to more projects than the smaller ones, and that the old member states are more frequently involved in projects than the newer member states. Regarding the institutions organisations participating in the EU funded projects ( Figure 14), the old member states (including the former member, the UK which has 7 of the top-25 institutions) are represented, whereas none of the newer member states have yet institutions among the 25 most frequent ones.

### Patents

The data on patents was extracted from PATSTAT using TIM and 3,428 existing patents on advanced nanomaterials were identified, of which 37 (1%) are related to smart nanomaterials. The search string used to extract the patents is shown in Table 2.

**Figure 15.**
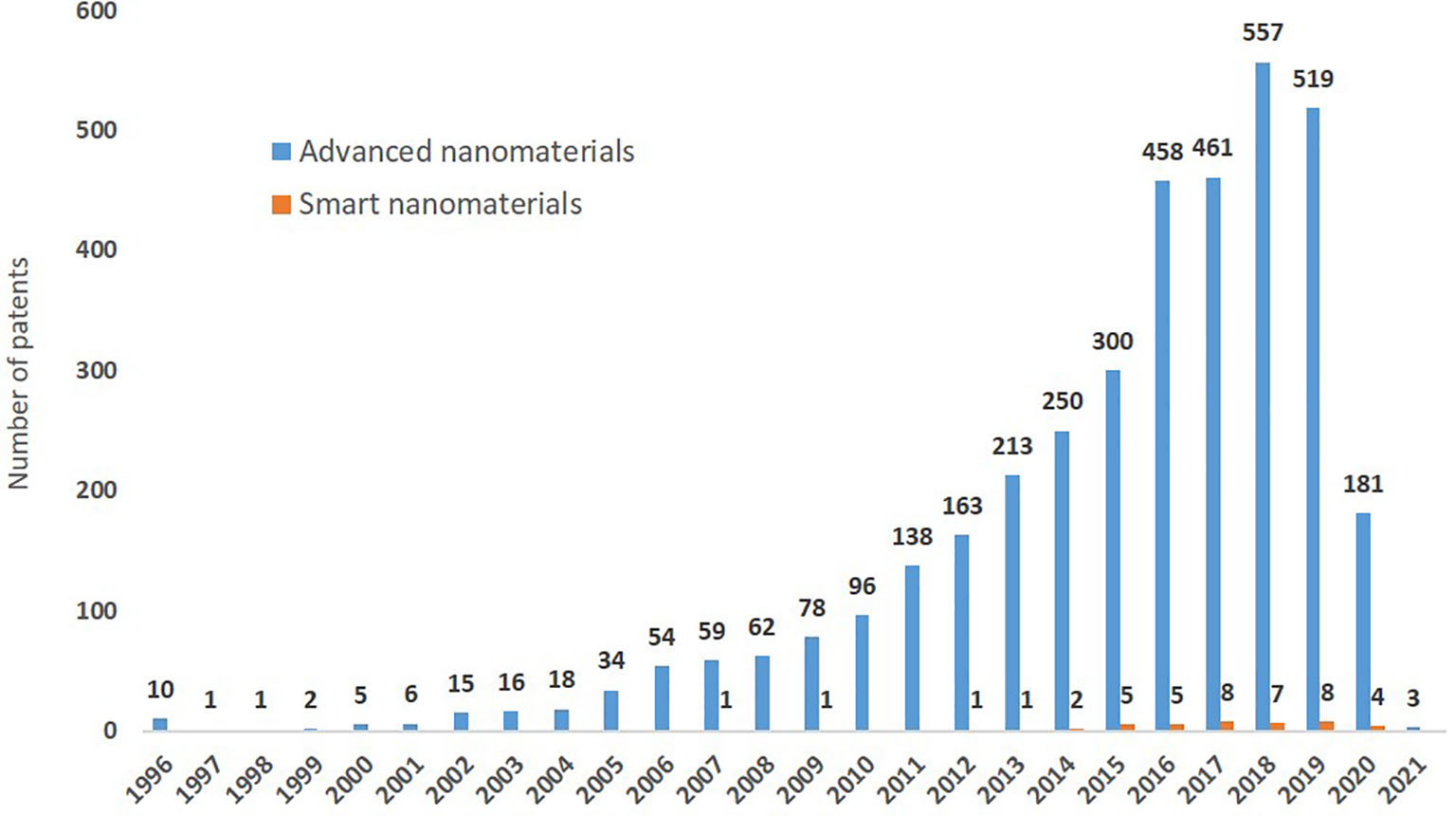
Yearly distribution of patents granted on advanced nanomaterials, including smart nanomaterials (orange).

When analysing the number of patents awarded for advanced nanomaterials per year in the period 2011 to 2021 ( Figure 15) there is a significant increase between 2011 and 2018 and a slight decrease in 2019-2020, which may just reflect the time that it takes to grant a patent. The first patent on smart nanomaterials was granted in 2007, and until 2013 at a maximum 1 patent per year was granted in this area, in 2014 two patents were granted, and afterwards 5 or more patents were granted each year. The small, but increasing, number of patents for smart nanomaterials may indicate that in the future, as this technology matures, more patents will be applied for and granted. The EC workshop ‘Safe and Sustainable Smart Nanomaterials’ ^32^ discussed a number of example areas in which smart nanomaterials are under development, for example agriculture ^41^^–^^46^ and medical applications. ^47^^–^^49^ These applications are still at the experimental research phase, but for agricultural applications they are clearly moving towards being the new way of dosing, in a timely manner and exactly needed amounts, essential nutrients and fertiliser as well as chemicals protecting plants against stressors. For medical applications smart nanomaterials are perceived for example as a future way of delivering medicine within the body to the precise location where it is needed. It is challenging to develop such applications into fully functional ones and time is required to go from identifying the concept to being able to patent it.

According to the International Patent Classification (IPC) ^50^ and USPTO Classification, ^51^ there are several areas of technology to which patents on advanced nanomaterials, including smart nanomaterials, pertain ( Table 4).

**Table 4.** Distribution of patents technology areas for advanced nanomaterials, including smart nanomaterials, according to the international patent classification.

Symbol	Area of technology	Number of sub-areas represented	Number of patents	Percent (%) of patents	Year of the first patent
A	HUMAN NECESSITIES	3	137	2%	1996
B	PERFORMING OPERATIONS; TRANSPORTING	20	1963	35%	1996
C	CHEMISTRY; METALLURGY	45	2030	36%	1996
D	TEXTILES; PAPER	-	-	-	-
E	FIXED CONSTRUCTIONS	-	-	-	-
F	MECHANICAL ENGINEERING; LIGHTING; HEATING; WEAPONS; BLASTING	-	-	-	-
G	PHYSICS	6	168	3%	2002
H	ELECTRICITY	20	775	14%	2007
Y	GENERAL NEW TECHNOLOGICAL DEVELOPMENTS *	6	535	10%	1996

* *Category according to the United States Patent and Trademark Office (USPTO): Y = general tagging of new technological developments; general tagging of cross-sectional technologies spanning over several sections of the IPC.*

**Figure 16.**
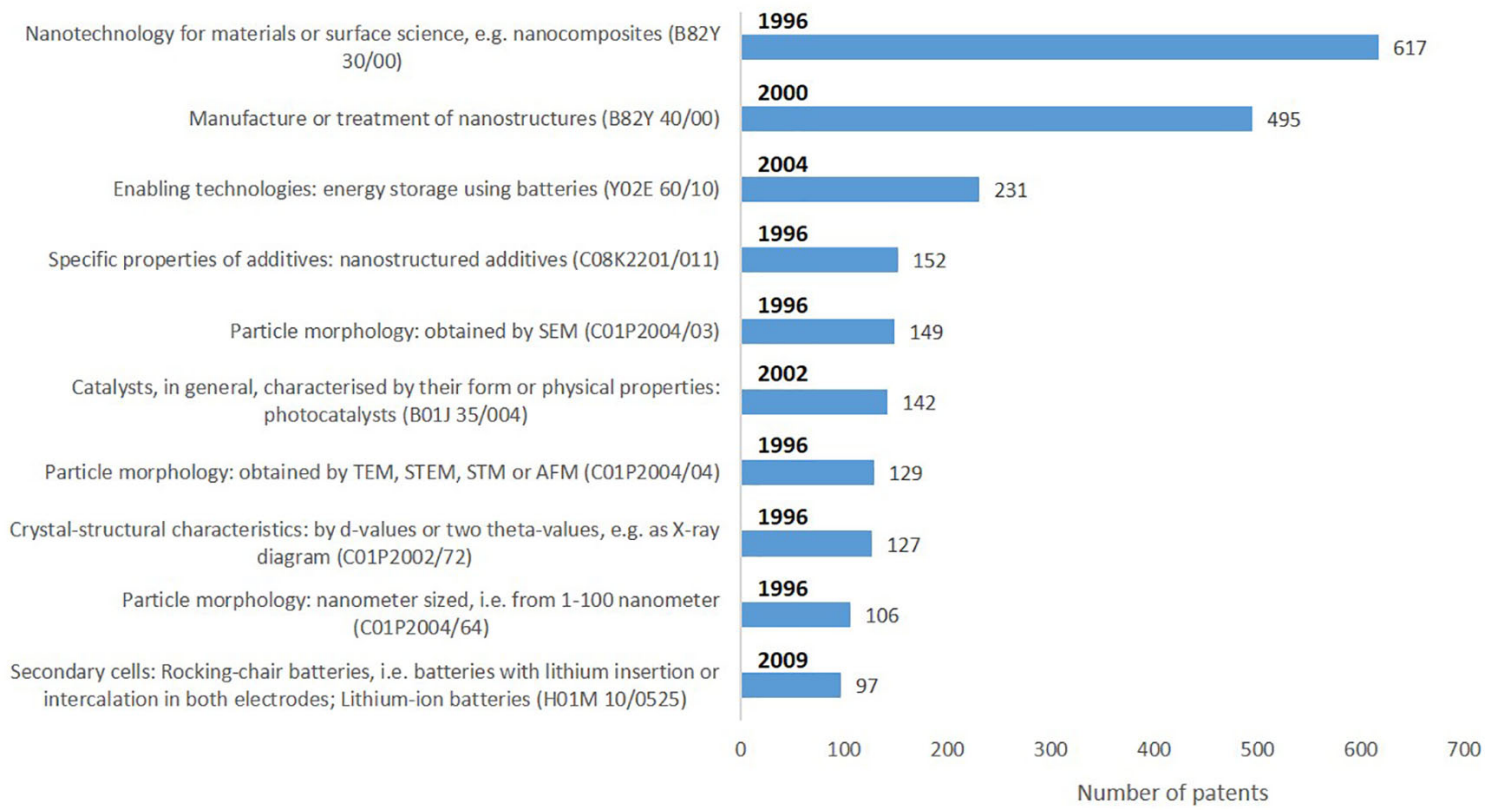
The ten most frequently assigned technology sub-areas for the patents on ‘advanced nanomaterials’ and number of patents granted for each of the sub-area represented.

**Figure 17.**
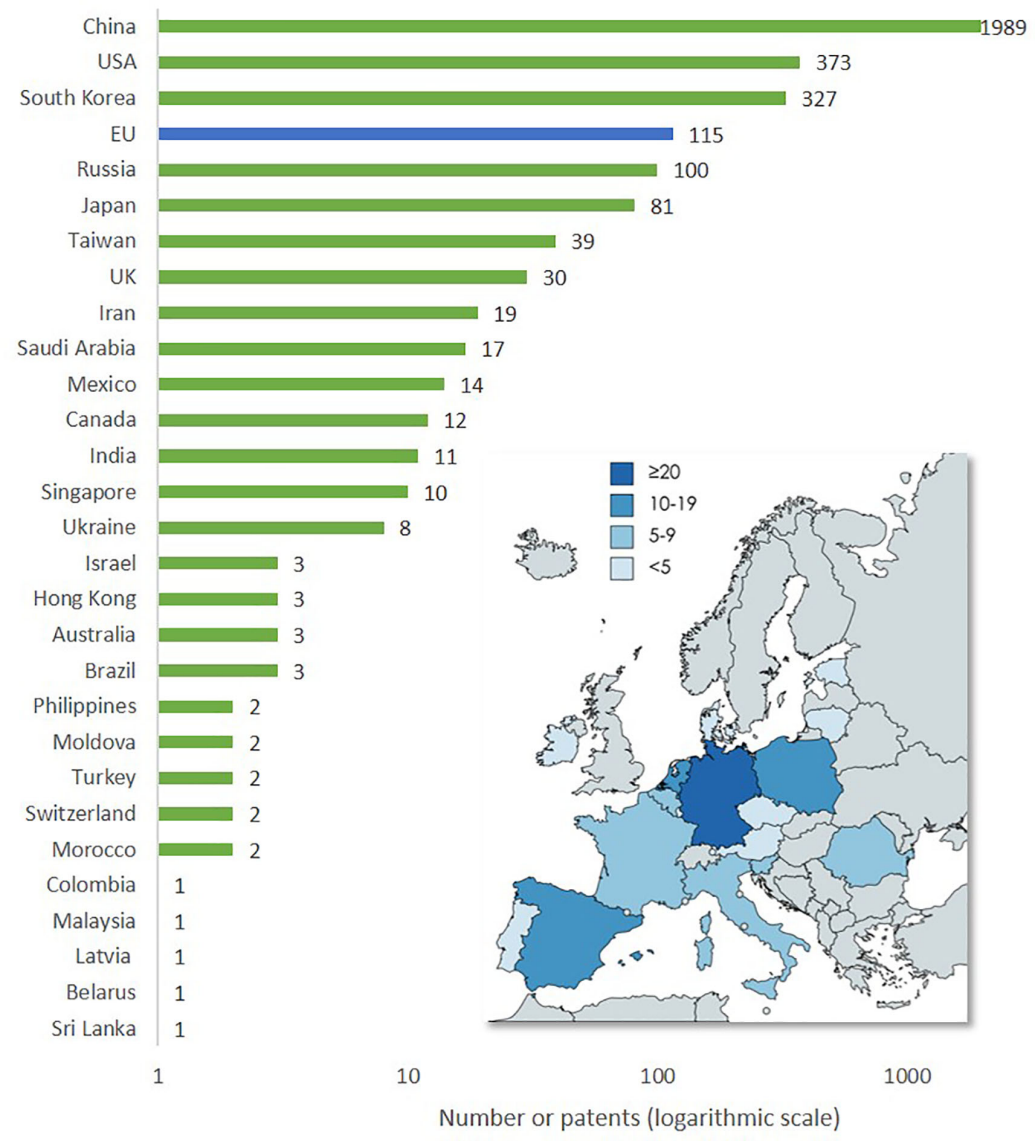
Global geographical distribution of patents on advanced nanomaterials (top 25 countries). The map shows the distribution of patent applications for advanced nanomaterials in EU countries.

An analysis ( *not illustrated*) of the technology areas of patents on ‘advanced nanomaterials’ versus the first year of using that technology area showed that in the period 1996 to 2021 the technology areas grew from 14 (in 1996) to 100 (in 2020), which is an indication of the uptake of advanced nanomaterials across innovative industrial applications. In the period of 1996 to 1999 the same 14 technology areas were considered, and more technology areas were used, adding between 495 patents (technology area applied since year 2000) and 20 patents (technology area applied since year 2007) to the number of patents granted. The indicators below ( Figure 16) shows the most frequent technology sub-areas to which the patents were assigned, as well as the geographical distribution of patents on advanced nanomaterials ( Figure 17).

## Discussion

### Key research and innovation indicators

*Current trends related to the advanced nanomaterials*

**Figure 18.**
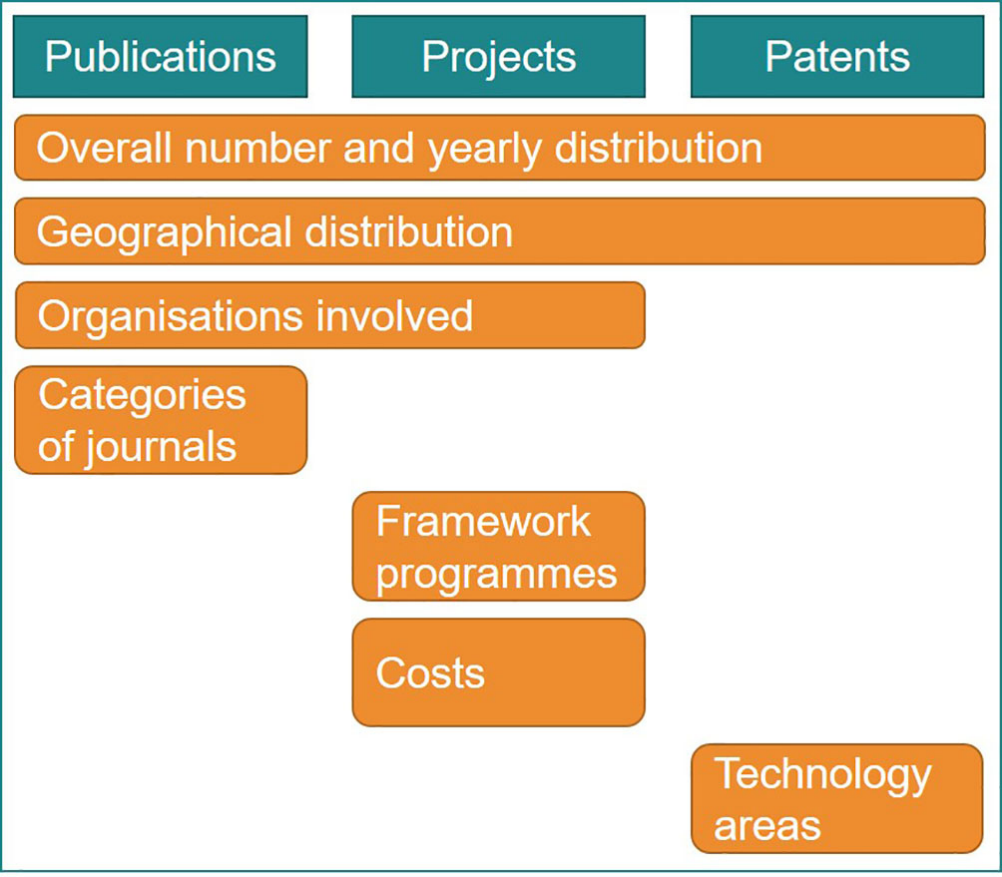
Indicators for publications, projects and patents.

The presented analysis aimed to build indicators for advanced materials allowing to identify areas of growth. Figure 18 below illustrates the top level of information extracted by the analysis. Based on trend analysis of publications, projects and patents, and applying a set of keywords identified through iterative analysis of policy documents, we gained an overview of the overall number of publications and their yearly distribution within each category (see Table 2), as well as their geographical distribution (see Figure 10). For publications, we identified the most frequently involved countries worldwide ( Figure 10). For the EU, we identified the number of publications on advanced nanomaterials per EU member state, the participation of each EU member state for projects (see Figure 13), as well as the top 25 organisations participating in EU projects (see Figure 14) related to advanced nanomaterials, which include organisations based in Switzerland (which is associated to the EU research programme) and the UK (which was deeply involved before Brexit in 2020). For publications only and the categories of journals in which they appeared see Figure 8. For the projects, the analysis focussed on the EU, and we elucidated information on the framework programmes and the costs of the projects ( Figure 12). The analysis of the patents provided information on the technology areas in which new patents were granted.

According to the ‘Science, research and innovation performance of the EU 2020’, ^16^ the EU accounts for about one fifth of the world’s R&D, publications and patents. As illustrated by the outcomes of the analysis of scientific publications, projects and patents on advanced nanomaterials, there has been a significant growth in research into advanced nanomaterials, including smart nanomaterials, in the period of 2012 to 2021 leading to an increased availability of information; see e.g. Figure 7 ‘Scientific publications’ cover peer-reviewed articles, reviews, book chapters and conference proceedings. EU’s funding schemes have increasingly supported research into advanced nanomaterials, including smart nanomaterials, see e.g. Figure 11; this in turn has contributed to increasing the number of scientific publications originating from within the EU. Also, the number of patents granted per year has increased significantly in the 10-year period analysed as shown in Figure 15.

Based on the information presented above it is evident that in the period 2011 to 2021 the number of scientific publications and patents have increased significantly, whereas the increase in the number of EU-funded projects has been less notable. However, the project funding increased from €464 million under FP7 (2007 to 2013) to €589 million under H2020 (2014 to 2020), or in other words from €66.3 million/year to €84.1 million/year (see Figure 12). Hence, on average each project would be bigger under H2020 than under FP7.

*Relevancy of indicators*

These indicators would lead to identification of trends of future scientific and technological achievements in the area of advanced nanomaterials, which in turn would be one information element when examining possible impacts on society and policy implications associated to these areas. For example, taken collectively this information reflects that the new technology is maturing and evolving from being a new research field to becoming applied science, and products containing the technology are becoming available also to the general public. However, the indicators in general but also those proposed here should be seen as a dynamic tool, as the input data may change every day (e.g. when new articles are published) but also regarding the keywords applied in the search queries to select the input data. The keywords used in this study are considered as representative terms for the area of advanced nanomaterials and the indicators proposed reflect the search string applied. As new descriptions will be available for advanced nanomaterials, they can be added to the list or other terms can be withdrawn from the string.

In addition, more detailed analyses can be performed in order to look closer at and analyse, for example the distribution of advanced nanomaterials per sectors (agriculture, construction, electronics, energy, environment, medicine, biotechnology, etc.), specific applications and use of advanced nanomaterials (fertilisers, paintings, cosmetics, packaging, textiles, bioelectronics, sensors, batteries, solar cells, water treatment, biomarkers, coatings, drug carriers, tissue engineering, 3D printing, optics, etc.), most visible types of advanced nanomaterials (frequently used, characterised, etc.), new (smart) materials and applications that are at R&D stage and may soon be placed on the market, production and consumption patterns, online searching trends by the general public, economic and social impacts, or educational and training programmes related to the area of advanced (nano) materials.

As mentioned, such indicators would need periodical updating of the input data and their timelines in order to maintain their relevance. As the description or definition of advanced materials might change over time, any indicator needs to be a dynamic tool being able to capture new developments in this area.

### Future of scientific and technological achievements in the area of advanced nanomaterials

The methodology used in the study by Giese *et al*. 2020 ^29^ for identifying publications containing keywords on advanced materials included searching with a set of keywords in the Web of Science Core Collection ^52^ and extracting the data published between 2000 and 2018. The following terms were identified and used for characterising materials regarding their functionality, structure and manufacturing processes:

• **Functionality**: “active materials”, “smart materials”, “functional materials”, “multifunctional materials” and “adaptive materials”;
• **Structure**: “structural materials”, “structured materials”, “multistructural materials” and “artificially structured materials”;
• **Manufacturing**: “advanced manufacturing” and “advanced processing”.

In our study, the starting point is the above-mentioned terms, which have been further refined and adapted, e.g. we have used keywords related to the ‘functionality’ and ‘structure’, applying them in the same search string. This approach has led to extraction of data (publications, projects and patents) that contain all possible combinations of the two characteristics (function and structure) of the advanced (nano) materials.

Often, advanced materials are at the nanoscale or have one or more nanoscale entities as components of their structure, for example ‘nanohybrids’. ^18^^,^^53^ In the case of nanostructured advanced materials, the assembly method is also reported ^54^ to play a role in determining their unique properties. In particular, smart nanomaterials both present more complex structures than conventional nanomaterials and are designed to have higher dynamism by actively transforming in response to external stimuli.

The Smart Nanomaterials Industry Analysis by BIS Research ^55^ forecasts the market to grow at a significant Compound Annual Growth Rate (CAGR) of 33% on the basis of value during the forecast period from 2019 to 2029. According to this study, North America dominated the global smart nanomaterials market with a share of 37% in 2019 and the key players were identified to be, in alphabetical order, Abbott, ANP Co. Ltd., Akzo Nobel N.V., Bayer AG, BASF SE, Clariant, Donaldson Company Inc., JM Material Technology Inc., Nanologica, Nanogate, NanoBeauty, OPTINANOPRO, The Nano Gard L.L.C., and Yosemite Technologies Co. Ltd.

### Possible impacts on society and policy implications

**Figure 19.**
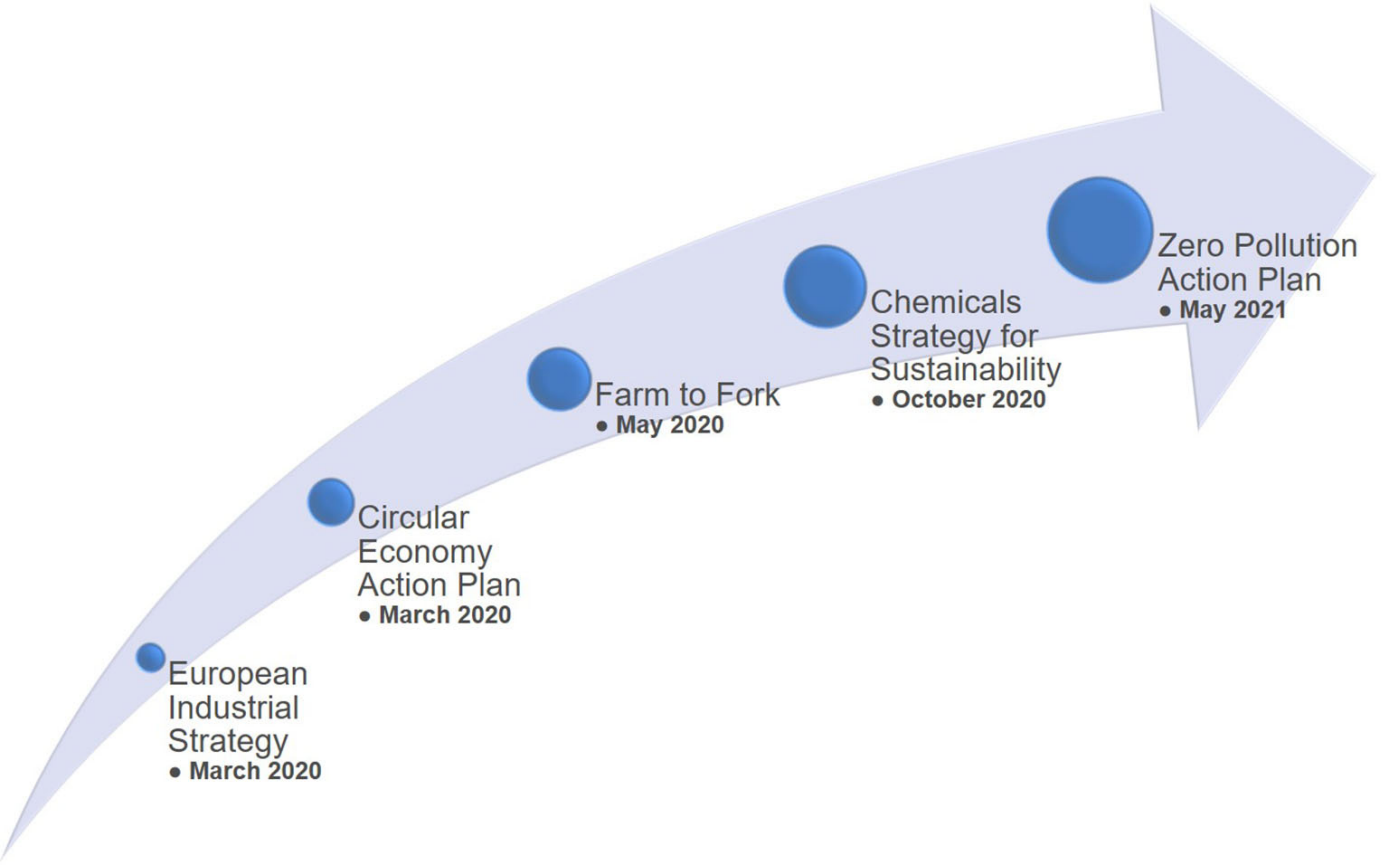
Communication of several actions related to the European Green Deal.

The EU policies related to the Green Deal ^6^ ( Figure 19), bring various opportunities to stakeholders (e.g. researchers, industry), but at the same time the policies come with major challenges regarding their implementation, e.g. upcoming updates (or new) of legislation, funding within the Horizon Europe Framework Programme (2021-2027) and beyond, and, in general, the alignment between legislation, industry and consumers. All these players will be influenced in one way or another by the implementation of the European Green Deal policy and its related actions (e.g. chemicals sustainability strategy, farm to fork, the industrial strategy and the circular economy action plan).

The Chemicals Strategy for Sustainability Towards a Toxic-Free Environment is presented in an EC Communication ^4^ that in its Annex proposes more than 80 key actions related to the implementation of the Chemicals Strategy for Sustainability. The actions are grouped in five main categories: *i)* innovating for safe and sustainable EU chemicals, *ii)* Stronger EU legal framework to address pressing environmental and health concerns, *iii)* Simplification and consolidation of the legal framework, *iv)* Providing a comprehensive and transparent knowledge base on chemicals and *v)* Provide a model inspiring chemicals management globally. In the coming years the actions will dominate the work on the EU chemicals policy, with a strong emphasis on updating the legislation towards a “ *stronger EU legal framework*”. According to the EC Communication, “ *the measures presented in this action plan, including legislative proposals and targeted amendments to REACH, will all need to be carried out in line with the better regulation principles and subject to evaluations and impact assessments as appropriate*”. One of these actions refer directly to nanomaterials (i.e. “Review of the definition of nanomaterial” which resulted in the adoption of the new definition ^56^), while the majority refer to chemicals in general and their safety and sustainability.

## Conclusions

**Figure 20.**
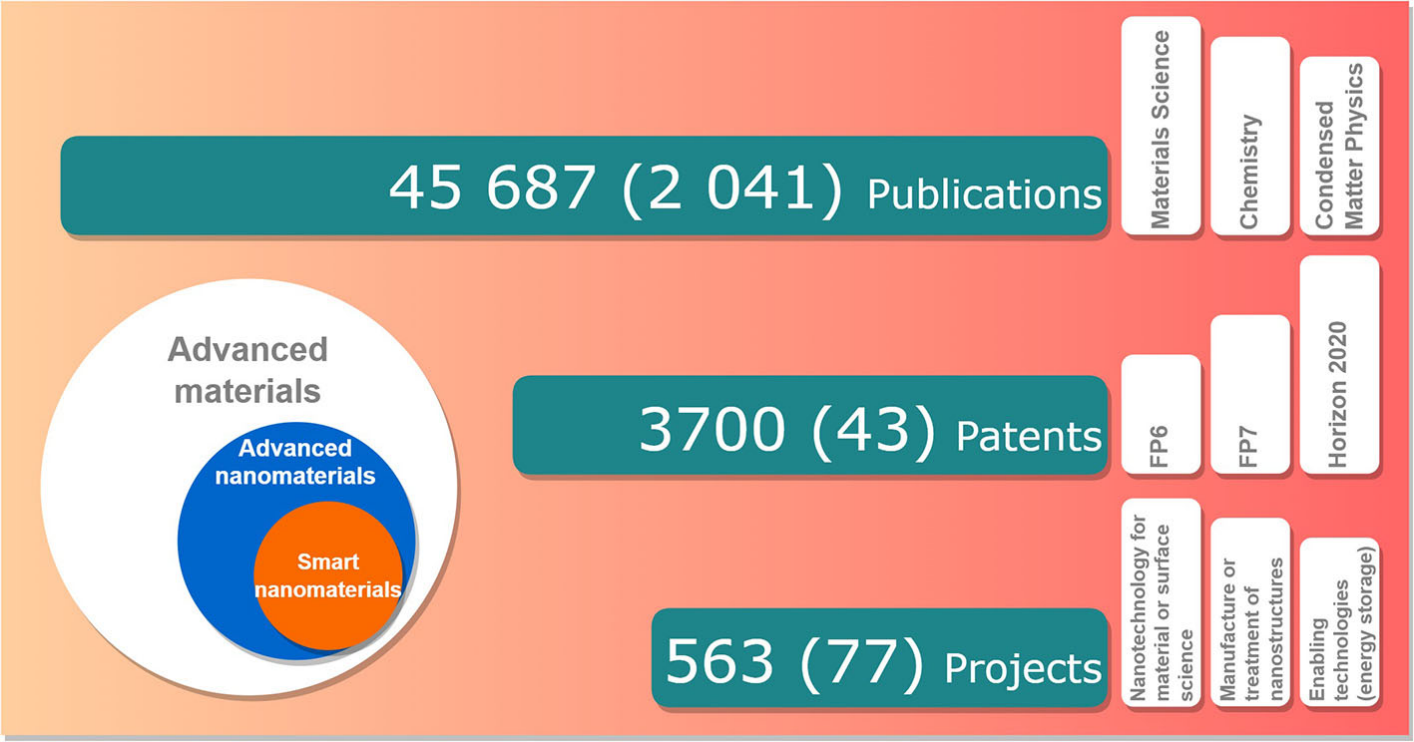
Summary results for the three fields analysed (1996-2021). The numbers represent the total items analysed for advanced nanomaterials, while the total number of items analysed for smart nanomaterials are given in the brackets.

The study established and tested the methodology for creating a set of research and innovation indicators in the area of advanced materials, with a focus on advanced nanomaterials and smart nanomaterials ( Figure 20):

• The methodology used a set of predefined keywords for data search and used several tools and databases for data extraction. The keywords are an essential element in establishing the indicators, as any modification will influence the input data used for the indicator. However, the authors consider that the set of keywords used in this study are representative and the results offer a good view on the analysed area. The methodology is reproducible provided the availability of access to the data extraction and databases mentioned in the study.
• The study developed a first set of indicators, in order to understand the level of complexity and data needed for such exercise and also to be used as a starting point for developing additional indicators or sub-indicators.
• Regarding the results, there is clearly an important growth both in scientific publications, patents and EU funding. Asia, led by China, is a very important player within the area of advanced materials (e.g. publishing and patents).

Several EU initiatives ^57^^–^^62^ are actively promoting ontology development, as well as the standardisation of documentation of data for advanced materials, including nanomaterials. The outcomes of such projects should further support the development of the classification of advanced materials as well as the progress of data interoperability and knowledge sharing. A clearer classification and description of advanced materials will also help building and refining indicators and similar tools for monitoring the development and impact in this area.

Indicators for identification and monitoring AdMa could feed into other initiatives, such as the Early4AdMa system ^63^ and can generally contribute to implementing a ‘Safer and Sustainable Innovation Approach’ (SSIA), currently developed by the OECD and being extended to integrate the safe and sustainable by design concept, ^64^ or support the risk screening for such materials, e.g. ^65^ Monitoring the area of AdMa could – with appropriate indicators to be developed – also provide timely insights into whether, how fast and how efficiently policy ambitions and action plans are turned into reality.

As a next step, the indicators can be further extended and developed (additional or more specific sub-indicators, identify new technologies or materials), while a dashboard that integrates all the indicators could be designed.

## Data Availability

The underlying data used for this publication were collected using the methodology presented above from the open sources databases ( SCOPUS, CORDIS, PATSTAT and Data.europa.eu) using open access data mining tools TIM Technology Editor ^40^ and Semantic Text Analyzer (SeTA). ^37^ No files were compiled but were temporarily exported from TIM (Microsoft Excel format) in order to generate the charts presented in the Results section. The additional analyses are performed directly in TIM and in order to reproduce the study, the data should be collected and analysed in TIM using the method presented above (e.g. by using the search string presented in Table 2).

## Footnotes

^1^

*Search string (data retrieved: 13 July 2022): topic:(nanoform OR nanomaterial) AND class:(conf OR boch OR review OR article) AND emm_year:[2012 TO 2021]*
