# Supplementary material for: Advanced materials foresight: research and innovation indicators related to advanced and smart nanomaterials
Source: F1000Res. 2023 Dec 28;11:1532. Originally published 2022 Dec 19. [Version 2] doi: 10.12688/f1000research.127810.2 (PMC10924728; doi:10.12688/f1000research.127810.2)
Supplement: Peer review history

# Reviewer report: Reviewer response for version 2

**DOI:** 10.5256/f1000research.160418.r248351

**Published:** 2024-03-19

**Authors:** Abdalla M Abdalla

**Affiliations:** 1 Mechanical Engineering Department, Faculty of Engineering, Suez Canal University, Ismailia, Ismailia Governorate, Egypt

I confirm that I have read this submission and believe that I have an appropriate level of expertise to confirm that it is of an acceptable scientific standard, however It is still needs some minor editing:

 1- The writings in the given tables must be in same font and as well as some figures needs to be increased in resolution.

 2- The author must provide SWOT analysis for this research topic and how nanomaterials will have different effects when applied in various fields. 

 3- Key challenges and limitations must be well discussed and clarified in the conclusion section, otherwise in a separate topic.

Is the work clearly and accurately presented and does it cite the current literature?

Yes

If applicable, is the statistical analysis and its interpretation appropriate?

Not applicable

Are all the source data underlying the results available to ensure full reproducibility?

Yes

Is the study design appropriate and is the work technically sound?

Yes

Are the conclusions drawn adequately supported by the results?

Partly

Are sufficient details of methods and analysis provided to allow replication by others?

Yes

Reviewer Expertise:

Materials Science, Energy Materials

I confirm that I have read this submission and believe that I have an appropriate level of expertise to confirm that it is of an acceptable scientific standard.

# Reviewer report: Reviewer response for version 2

**DOI:** 10.5256/f1000research.160418.r248353

**Published:** 2024-03-09

**Authors:** Priscila Falagan Lotsch

**Affiliations:** 1 Department of Biological Sciences, College of Sciences and Mathematics, Auburn University, Auburn, Alabama, USA

The manuscript aims to pinpoint areas of scientific research and technological development of advanced materials, specifically focusing on advanced nanomaterials and smart nanomaterials. The study adopted an iterative approach, beginning with identifying and collecting data to establish relevant research and innovation indicators. Data were collected from reputable databases such as SCOPUS, CORDIS, and PATSTAT, using predefined search strings tailored to capture relevant information on advanced nanomaterials. The analysis encompassed trends, distributions, and other pertinent aspects, including geographical distribution, organizational involvement, research areas, and funding programs. The manuscript is well-written and clear and provides valuable insights into trends, impacts, and implications of advanced nanomaterials. The study contributes to a better understanding of the future landscape of advanced materials, and the findings presented serve as a foundation for further evaluations of potential societal impacts and policy implications, ultimately guiding stakeholders toward informed decision-making and responsible innovation in the field of advanced nanomaterials.

Is the work clearly and accurately presented and does it cite the current literature?

Yes

If applicable, is the statistical analysis and its interpretation appropriate?

Not applicable

Are all the source data underlying the results available to ensure full reproducibility?

Yes

Is the study design appropriate and is the work technically sound?

Yes

Are the conclusions drawn adequately supported by the results?

Yes

Are sufficient details of methods and analysis provided to allow replication by others?

Yes

Reviewer Expertise:

Nanomaterials, Nanotoxicology, Nano-Bio Interactions

I confirm that I have read this submission and believe that I have an appropriate level of expertise to confirm that it is of an acceptable scientific standard.

# Reviewer report: Reviewer response for version 1

**DOI:** 10.5256/f1000research.140352.r183340

**Published:** 2023-09-05

**Authors:** Ander Reizabal

**Affiliations:** 1 Basque Center for Materials, Applications and Nanostructures, University of Oregon, Eugene, Oregon, USA

This study undertakes the pivotal task of discerning the landscape of scientific research and technological advancements within the realm of advanced materials, with a specific emphasis on advanced nanomaterials, particularly the sub-category of smart nanomaterials. Its primary objective revolves around the systematic collection and meticulous analysis of data to construct relevant research and innovation indicators, thereby providing profound insights into prevailing trends, impacts, and other significant facets within this dynamically evolving field. To ensure clarity and transparency, this paper expounds upon the methodologies employed, encompassing an iterative process that involves documentation, the establishment of core research and innovation indicators, and the utilization of prominent databases, including SCOPUS, CORDIS, and PATSTAT.

 However, as we embark on this scholarly journey, it is essential to address some critical aspects and potential refinements within the study's foundations and methodologies:

**Methodology Foundations:**

1. Objective Clarity: While the study's intent is to provide an objective representation of reality through data analysis, the introductory section contains repeated expressions and sentences that may not align with the desired objectivity. Enhancing the clarity of the introduction will effectively communicate the transparency and objectivity of the authors.
2. Definition of Advanced Materials: The definition provided for "advanced materials" is broad and subjective, leaving room for interpretation. Defining what constitutes an "advance" in materials science is vital to avoid ambiguity.
3. Keyword Selection: The selection of keywords, as illustrated in Figure 3 and the related text, warrants meticulous consideration. Each chosen term should be critically evaluated for its relevance and significance within the context of advanced materials. The rationale behind their selection should be clearly elucidated.
4. Relevance of Keywords: The mere presence of a keyword in a paper does not necessarily imply a substantive connection to the topic. The study should explore methods to determine the relevance and significance of each keyword in the context of the research.
5. Sources of Innovation: It is important to acknowledge that reviews and books often compile existing research rather than presenting novel findings. This could potentially result in the duplication of research efforts. Additionally, the mere inclusion of a keyword in a project or conference presentation may not indicate innovation; it should be considered in the context of project outcomes.

**Non-Methodology Points for Discussion and Inclusion:**

1. Comprehensive Data: Figures such as 10, 13, 14, 15, 16, and 17 provide valuable insights but could benefit from a broader perspective. Incorporating data related to the overall growth in relevant fields can provide context and enhance the interpretation of specific trends.
2. Grant Outcomes: Exploring data on the success rates and outcomes of grants related to advanced materials could be enlightening for stakeholders. Identifying which grants have led to tangible advancements or commercial products would be particularly valuable.

**Discussion and Conclusions:**

1. Maturity of Technology: The statement regarding technology maturation evolving from a research field to applied science requires a more robust foundation. The study should elaborate on the evidence supporting this assertion.
2. Keyword Representation: The claim that the chosen set of keywords is representative and provides a comprehensive view of the analyzed area needs substantiation. Discussing the methodology's robustness and its ability to yield a comprehensive representation would enhance confidence in the study's results.

In conclusion, this study marks an important step toward understanding the landscape of advanced materials, but careful consideration and refinement of its foundations, methodologies, and interpretations will enhance its relevance and credibility within the field of materials science research.

Is the work clearly and accurately presented and does it cite the current literature?

Yes

If applicable, is the statistical analysis and its interpretation appropriate?

Not applicable

Are all the source data underlying the results available to ensure full reproducibility?

Yes

Is the study design appropriate and is the work technically sound?

No

Are the conclusions drawn adequately supported by the results?

No

Are sufficient details of methods and analysis provided to allow replication by others?

Yes

Reviewer Expertise:

Materials science, smart materials

I confirm that I have read this submission and believe that I have an appropriate level of expertise to confirm that it is of an acceptable scientific standard, however I have significant reservations, as outlined above.

# Response

**Published:** 2023-12-19

**Authors:** Lucian Farcal

**Affiliations:** European Commission - Joint Research Centre, Italy

Thank you very much for reviewing the paper and for the suggestions, which we have addressed in the second version of the article. Several sections were refined in order to reflect the comments received, e.g.:

- The Introduction, including the ‘study objectives’ was refined.
- The Methodology section was further refined to bring more clarity, especially related to the data collection and analysis.
- The Results section was refined. The data shown has not been modified.

**Methodology Foundations:**

**1. Objective Clarity**

 The introduction section starts with the EU and international context of the study by referring to the EU policy context and ongoing activities in this area at international level (e.g. OECD) or as part of different research projects. These activities proposed already a set of definitions and classifications for advanced materials which were used as a base for the study and further extraction of data for the indicators. As such, the introduction aimed to list the essential policy and technical aspects needed in this context.

 In this context (and as part of a larger foresight study) the objectives were designed to address a first set of aspects related to research and innovation in this area, reflected in research projects, publications and patents.

**2. Definition of Advanced Materials**

 Defining the advanced materials was outside the scope of this study, therefore the authors exploit existing resources. The starting point of the study were currently agreed definitions by the scientific community, of advanced materials. These generated a set of representative terms, used later as keywords for extracting data.

 However, we fully agree that defining what is ‘advanced material’, is crucial for such a study and the authors tried to cover these aspects in the introduction and further in the Discussion section.

 Perhaps it is important to clarify also that the study did not intend to address specific components of advanced materials (that could be included in future and extended indicators).

**3. Keyword Selection**

 Figure 3 was generated automatically with an online tool (Semantic Text Analyser - SeTA) that applies advanced text analysis techniques to large document collections, methodology detailed in the JRC Technical Report (Reference 38). SeTA was used as complementary tool for the selection of representative keywords, for the refinement and validation of previous background analysis.

**4. Relevance of Keywords**

 The authors agree that additional methods may exist and can be applied for increasing the relevance and specificity of data extracted. The methodology was developed in an iterative manner, with the aim of identifying a set of ‘representative keywords’, without the intention of being complete. The authors consider that the keywords used are highly significant (considering the definitions used), while the relevance is adequate but indeed, both aspects can be further refined e.g. in case more specific sub-indicators will be developed.

 Acknowledging that the reviewer’s observation (The mere presence of a keyword in a paper does not necessarily imply a substantive connection to the topic.) is fully correct, we note that the study extracts statistical data, and hence will give an overall impression of where the field is going and that individual papers could be off the mark or undetected.

**5. Sources of Innovation**

 For the core indicators presented, the authors preferred not to discriminate between the types of publications. We agree that additional, more specific, sub-indicators can be created using only a sub-set of data (e.g. categories of data shown in Figure 6). Such aspects were not within the scope of the current study, which intended to cover more general indicators.

**Non-Methodology Points for Discussion and Inclusion:**

**1. Comprehensive Data**

 We thank the reviewer for sharing this idea. This would generate more complex, composite indicators, which could be one of the follow-ups of this study that aimed to generate a set of simple indicators using the tree main databases mentioned in the Methodology.

**2. Grant Outcomes**

 We recognise the importance of this aspect and note that it could be further explored in a follow-up study, as it could not be addressed in the current study.

**Discussion and Conclusions:**

 We thank the reviewer for this positive feedback. Discussion section has been refined.

# Reviewer report: Reviewer response for version 1

**DOI:** 10.5256/f1000research.140352.r158446

**Published:** 2023-01-11

**Authors:** Steffi Friedrichs

**Affiliations:** 1 AcumenIST SPRL, Etterbeek, Belgium

The paper is a well-written piece of a timely study into the keywords related to the field of advanced materials and nanomaterials (i.e. including 'smart' nanomaterials'). The paper furthermore represents and excellent example of how policymaking could and should be connected to and supported by scientific evidence.

 In the following points of mild 'criticism', I would like to encourage an improvement of the paper over and beyond its readily publishable nature.

1. A bit of more generous punctuation or rephrasing could support better readability:
  - 'The EU has e.g. the largest share of worldwide patent applications in advanced manufacturing technologies and the Internet of Things for mobility.' - this sentence worked in spoken language, but not in written form. the same is tru here: 'Regarding smart nanomaterials e.g. “ *It is expected that future research activities in the European Union will investigate whether the current approach to safe-by-design covers the dynamic features of smart nanomaterials too and, if not, how to adapt it and provide manufacturers and regulators with the appropriate tools for its implementation.*”'
2. detailed list of typos / errors:
  - a space missing in the caption of Figure 9
  - in the brackets of the first sentence under the header ‘Advanced and smart (nano)materials’: ‘or the products and processes in which they are used for’ (delete either ‘in’ or ‘for’.

 

 The figures could be improved, and their captions made clearer:

- Figure 1: it is not correct that 'advanced materials' necessarily lies on a direct line between 'European Green Deal' and 'tools and data', because the latter's sub-sections are directly related to each other. A triangular arrangement of the green, blue and red clusters may be more appropriate. Moreover, the caption should be more detailed (i.e. describe what the small hexagons around the big central ones are).
- Figure 2: the caption needs to be more detailed: what do the numbers (1) - (2) - (3) describe. The figure should take more care to making sure that the elements of a list are of comparable nature (i.e. 'consumers' and 'environment' in a list of 'policy', 'legislation', 'funding')
- Figure 8: there needs to be a note explaining, which sort of categorisation was used (i.e. where it comes from). Most importantly, many journals are assigned to more than one category; it needs to be stated that the statistics were correct for this.
- Figures 11 and 12: the data on Projects is the most wobbly, when it comes to key-word analyses, because projects are based on their underlying proposal text, which are written for an entirely different purpose than publications and patents are written for: proposals aim to leverage funding during a stringent evaluation process and on a limited number of pages. There are a number of limitations and biases that should be taken into account when using text-mined data from projects; these include:
  - The statistics used here capture winning proposals only.
  - the terms used in a proposal (and thus) project text are likely to mimic/copy those used in the call text (where projects are bound to call text); this was one of the finding in our text-mining analyses for the NanoFabNet Project (https://acumenist.com/mapping-the-scientific-landscapes-of-nanofabrication-sustainability/) [NOTE: we were alerted to this 'bias' by finding that the keywords found in 'open call'-projects (e.g. Marie Curie) seemed to correspond more closely to those of publications, while the keywords used in 'call-text-bound'-calls seemed to be lagging behind in the use of keywords (because they keywords would first need to be included in the next multiannual framework, before they are used in successful proposals)].
  - similar to the previous bullet, the amount of funding for a specific area depends mostly on how much money had been earmarked for a specific scientific field by the EU.
- Figure 13: it does not seem appropriate to exclude the UK from the visualised result for the simple reason that UK-institutes are included in Figure 14. The same underlying selection should be applied to both figures so as to render them comparable.
- Figure 15: there should be a note explaining the drop of patents at 2020 and 2021; this is an artefact that is to do with the handling and publication of patents through the relevant databases; since this artefact does not apply to publications or projects, it needs to be explained to keep the data comparable.

 

 Specific comment/suggestion:

 - should 'actuator' not be one of the key-words (see table 1)

 Comment regarding the conclusions:

 Tt would be desirable to draw more possibilities/recommendations from the conclusions. The text makes a link between the KPIs that the EC wants to develop and the CSS, but how will the analysis and results presented in this paper contribute to this?

 The paper says *'These indicators would lead to identification of trends of future scientific and technological achievements in the area of advanced nanomaterials, which in turn would be one information element when examining possible impacts on society and policy implications associated to these areas.'* and it would be interesting to read more about 'how exactly' this can be achieved.

Is the work clearly and accurately presented and does it cite the current literature?

Yes

If applicable, is the statistical analysis and its interpretation appropriate?

Partly

Are all the source data underlying the results available to ensure full reproducibility?

Yes

Is the study design appropriate and is the work technically sound?

Yes

Are the conclusions drawn adequately supported by the results?

Yes

Are sufficient details of methods and analysis provided to allow replication by others?

Yes

Reviewer Expertise:

nanotechnology, chemistry, materials, sustainability, innovation analysis and managament (incl. EU projects), standardisation, policy analysis, policy development, statistical analyses (incl. text-mining)

I confirm that I have read this submission and believe that I have an appropriate level of expertise to confirm that it is of an acceptable scientific standard.

# Response

**Published:** 2023-03-02

**Authors:** Lucian Farcal

**Affiliations:** European Commission - Joint Research Centre, Italy

Thank you very much for reviewing the paper and for the useful suggestions on both the content and formatting, that we hope are reflected in the second version of the article ( to be uploaded).

**1. **The document was proofread once again and the text refined, as indicated and as needed, in order to improve its readability. Please note that some sentences indicated are ad verbatim citations of other papers, and we have thus not changed.

**2.** Typos were corrected.

**3. Figures**:

- **Figure 1**: the format was changed as suggested. The caption was completed with an additional explanation regarding the second layer of hexagons.
- **Figure 2**: the caption was extended with an explanation on the numbers (they are linked to the study objectives). Regarding the elements included together in the right-side box, in order to distinguish between them, they were represented in different colours. However, for this schematic representation of the objectives, the comparability between the main impacted areas was not considered essential, but rather their identification.
- **Figure 8**: the categorisation used is based on the categories defined by the SCOPUS database (see Scopus Subject Areas), from where the original data was extracted. Indeed, one journal (and its publications) may be assigned to more than one category. This is now mentioned in the text under Methods (under ‘Data extraction and analysis’ section) as well as in the Results, in the text related to Figure 8.
- **Figures 11 and 12**: we are indeed aware of the limitations of automatic data mining methods generally for such purposes and also in this particular case: 
  - Indeed, the indicator represents only funded (winning) projects, as mentioned in the text. 
  - The tool used for data mining, TIM, uses the public information displayed in CORDIS and that is listed under the ‘Fact Sheet’ of the projects (title and abstract). The full proposal, generally, is not publicly available and cannot be searched. It would be interesting to look also in the Reporting or Results sections of the projects in order to cover not only the proposed text, but also the outcomes. For the latter, some results should appear in the publication statistics. However, such detailed analysis was not under the scope and was not performed in this study. 
  - Additional details on the search methodology were included in the text (under “Data extraction and analysis”) 
- **Figure 13**: this figure is based on TIM’s automatic filter for EU countries and aimed to reflect the situation at the moment when the data was extracted (2022), for EU. In addition, the analysis looked in more detail, in order to show also the contribution of former EU countries (such as UK) or associated countries in the Horizon FPs (such as Switzerland), etc., through their organisations involved in the projects. A more general indicator (including all European countries or worldwide, not only EU) may be used to show this data.
- **Figure 15**: short additional text was added to the text related to this figure. (In general, the indicators are indeed a dynamic tool for which the data may change frequently, as the new resources are published and made available online). 

**4. Specific comment/suggestion**:

 Thank you for the suggestions. For this study, it will not be possible to add it to the current list, as the addition of a new keyword will influence the whole set of indicators, thus necessitating a full repetition of the study. It should also be noted that one of the aims was to create a first list of keywords relevant for advanced materials, which can be refined based on the feedback received and can be used in any other context. Hence, the list of keywords can be updated in the next phases of the study. 

 For example, the addition of ‘actuator’ to the search list, would add e.g. nine new (9) publications (only for 2019), six new (6) patents to the overall data and no new projects.
